# Effect of Transcranial Direct Current Stimulation Combined With Rehabilitation on Arm and Hand Function in Stroke Patients: A Systematic Review and Meta-Analysis

**DOI:** 10.7759/cureus.101404

**Published:** 2026-01-12

**Authors:** Baraah A Odeh, Faisal Alotaibi, Bayan H Khamis, Norah F Almugren, Ali S Aljazi, Mohammed S Lasloom, Reem D Alshahrani, Ebtisam M Almutairi, Muzun A Zafarani, Khulud F Alshammari, Shaima Alsamiri, Yazeed S Jabr, Saleh M Alhirsan

**Affiliations:** 1 Neurological Physiotherapy, Hashemite University, Zarqa, JOR; 2 Physical Therapy, Shaqra University, Shaqra, SAU; 3 Physiotherapy, Al-Baha Health Cluster, Al-Baha, SAU; 4 Physical Therapy, Maternity and Children's Hospital - Tabuk Health Cluster, Tabuk, SAU; 5 Physical Therapy, College of Applied Medical Sciences, King Saud University, Riyadh, SAU; 6 Musculoskeletal Physiotherapy, Najran Health Cluster (Habuna General Hospital), Najran, SAU; 7 Physical Therapy, King Khalid University, Abha, SAU; 8 Physical Therapy and Rehabilitation, Ministry of Health - Saudi Arabia, Riyadh, SAU; 9 Physical Therapy, Prince Mansour Military Hospital, Taif, SAU; 10 Physical Therapy, Ministry of Health - Al Jouf, Sakaka, SAU; 11 Physical Therapy, Umluj General Hospital, Umluj, SAU; 12 Physiotherapy, College of Applied Medical Sciences, Umm Al-Qura University, Makkah, SAU; 13 Physical Therapy and Health Rehabilitation, College of Applied Medical Sciences in Quarayyat, Jouf University, Sakaka, SAU

**Keywords:** meta-analysis, motor recovery, rehabilitation, stroke, transcranial direct current stimulation (tdcs), upper extremity

## Abstract

Stroke is a major cause of chronic disability, as motor deficits in the upper limbs remain a persistent issue for many survivors. Transcranial direct current stimulation (tDCS) is a potential non-invasive neuromodulation method to enhance neuroplasticity, but evidence regarding its effectiveness when paired with rehabilitation remains mixed. This systematic review and meta-analysis aimed to assess the impact of adding tDCS to rehabilitation on upper limb motor recovery and activity. Electronic databases were searched for randomized controlled trials (RCTs) comparing active tDCS (anodal, cathodal, or bihemispheric) plus rehabilitation against sham tDCS plus rehabilitation. The primary endpoint was motor impairment of the upper extremity, evaluated via the Fugl-Meyer Assessment for upper extremity (FMA-UE). Secondary endpoints comprised functional activity, grip strength, and safety profiles. A random-effects model was employed for data synthesis, and risk of bias was evaluated using the Cochrane RoB 2 tool. The analysis included 45 RCTs with 1,568 participants. The combination of active tDCS and rehabilitation demonstrated a statistically significant enhancement in FMA-UE scores relative to sham (mean difference (MD) = 7.11, 95% CI: 5.76 to 8.46; p < 0.0001), surpassing the minimal clinically important difference. Subgroup analysis revealed that the effect was most pronounced in the subacute phase (MD = 11.72) compared to the chronic phase (MD = 4.89). Functional activity also showed significant improvement (standardized mean difference = 0.62). No significant differences were found between stimulation montages (anodal, cathodal, bihemispheric). Adverse events were similar between groups. tDCS appears to provide a clinically meaningful adjunctive benefit to rehabilitation for improving upper limb motor function in stroke patients, particularly in the subacute phase. The findings support the potential integration of tDCS into stroke rehabilitation protocols, although future research should focus on optimizing stimulation parameters and personalizing treatment.

## Introduction and background

Stroke is a leading cause of enduring adult disability, with upper extremity dysfunction emerging as one of its most persistent and challenging consequences [1,2]. Many survivors contend with lasting hemiparesis, reduced manual dexterity, and compromised motor control, all of which limit functional independence and lower quality of life. Although standard neurorehabilitation approaches, such as task-specific training and constraint-induced movement therapy (CIMT), form the foundation of recovery, therapeutic results are frequently partial, leaving many individuals with significant residual deficits [1]. There is a critical demand for adjunctive therapies capable of enhancing neuroplasticity to improve the effectiveness of conventional physical rehabilitation.

Post-stroke motor deficits are often explained by the interhemispheric competition model. After a unilateral cerebrovascular event, the primary motor cortex (M1) on the affected side demonstrates lower excitability, whereas the unaffected M1 exerts excessive transcallosal inhibitory signals on the damaged hemisphere, stifling motor output [1,2]. Transcranial direct current stimulation (tDCS) is a valuable noninvasive brain stimulation (NIBS) tool for rebalancing cortical excitability. By applying weak electrical currents via the scalp, tDCS creates polarity-specific changes in neuronal resting membrane potentials; specifically, anodal stimulation enhances depolarisation and excitability, whereas cathodal stimulation promotes hyperpolarisation and inhibition [3,4].

In addition to temporary membrane polarization, tDCS supports synaptic plasticity via pathways analogous to long-term potentiation (LTP) and long-term depression (LTD), driven by glutamatergic transmission and the modulation of brain-derived neurotrophic factor (BDNF) [2,3]. Current biophysical theories propose that tDCS efficacy is state-dependent; rather than generating action potentials, the technique modulates the firing rates of neurons that are already active. This principle of functional targeting suggests that tDCS benefits are optimized when paired with neuronal activation from behavioral tasks [3,5]. Combining tDCS with active physical therapy may generate a synergistic effect, priming the motor cortex to respond effectively to motor learning [2].

Despite this strong physiological rationale, clinical evidence regarding the efficacy of tDCS in stroke recovery remains inconsistent. While some meta-analyses suggest moderate improvements in motor function, individual randomized controlled trials (RCTs) often report conflicting results regarding the optimal montage (e.g., bihemispheric vs. unilateral), timing (online vs. offline), and specific impact on functional activities versus impairment scales [1,5]. Furthermore, the translation of neurophysiological gains into meaningful improvements in Activities of Daily Living (ADLs), often assessed with patient-reported outcome measures such as the Motor Activity Log [6], remains a subject of ongoing investigation.

This systematic review and meta-analysis aimed to synthesize high-quality RCTs to determine the efficacy and safety of tDCS as an adjunct to upper limb rehabilitation in stroke patients. Specifically, this review evaluated the cumulative effect of combined tDCS and rehabilitation on reducing motor impairment, restoring functional limb capacity, and improving patient-perceived performance in ADLs. By analysing data across different stroke stages and stimulation protocols, this study seeks to clarify the clinical utility of tDCS and inform evidence-based guidelines for neurorehabilitation.

## Review

Methods

Protocol and Registration

This study adheres to the guidelines established by the Preferred Reporting Items for Systematic Reviews and Meta-Analyses (PRISMA) statement [7]. The review protocol was defined a priori and registered with the International Prospective Register of Systematic Reviews (PROSPERO) under the identification number CRD420251181685 [8].

Search Strategy

An exhaustive search of the literature was performed from inception up to the current date, restricting results to English-language publications. PubMed, MEDLINE, Embase (via Ovid), PsycInfo, Scopus, Web of Science, CINAHL, and the Cochrane Central Register of Controlled Trials (CENTRAL) were queried. The search strategy incorporated Medical Subject Headings (MeSH) and free-text terms relevant to “stroke”, “upper extremity”, and “transcranial direct current stimulation”. Boolean operators were used to combine search terms: "OR" was used to combine synonyms within a concept (e.g., Stroke OR Cerebrovascular Accident), and "AND" was used to link the three primary concepts (Population AND Intervention AND Region). The reference lists of included trials and prior systematic reviews were manually screened("snowballing") and checked clinical trial registries for unpublished data to maximize retrieval.

Eligibility Criteria

Selection was based on the PICOS framework [9]. RCTs were included, involving adult stroke survivors (aged ≥18 years) in any recovery phase (acute, subacute, or chronic). Eligible interventions used active tDCS (anodal, cathodal, or bihemispheric) administered concurrently or sequentially with upper limb rehabilitation (e.g., physical therapy, occupational therapy, robotics, or CIMT). Control groups must have received sham (placebo) stimulation alongside identical rehabilitation. The primary endpoint was upper extremity motor impairment, quantified by the Fugl-Meyer Assessment for upper extremity (FMA-UE) [10]. Secondary endpoints included functional scales such as the Action Research Arm Test (ARAT) [11], Wolf Motor Function Test (WMFT) [12], and Box and Block Test (BBT) [13], as well as safety metrics including dropout rates and adverse events.

Data Extraction and Management

Two independent reviewers extracted data using a standardised form. The extracted data fields included study characteristics such as author, year, sample size (N), and stroke chronicity (acute/subacute vs. chronic). The tDCS parameters were also recorded, including the electrode montage (target site), current intensity (mA), duration per session (minutes), electrode size (cm²), current density (mA/cm²), and total treatment duration (min).

The extracted outcome data included sample sizes, means, and standard deviations (SD) for post-intervention and follow-up time points. The number of adverse events and dropouts was recorded for safety. To avoid unit-of-analysis errors in studies with multiple active intervention groups sharing a single control group, data from active arms (for example, Anodal and Cathodal) were pooled into a single "Active tDCS" group before comparison with the Sham group, following the recommendations from the Cochrane Handbook for Systematic Reviews of Interventions [9].

Risk-of-Bias Assessment

The methodological quality was assessed using the Cochrane Risk of Bias 2 (RoB 2) tool [14]. Five domains were evaluated: bias arising from the randomization process, bias due to deviations from the intended interventions, bias due to missing outcome data, bias in the measurement of the outcome, and bias in the selection of reported results. Regarding the handling of missing participant data, values were extracted based on Intention-to-Treat (ITT) principles, as reported by the original authors; otherwise, available case analysis was performed based on the reported means and standard deviations of completers. Studies were classified as "low risk”, "some concerns”, or "high risk”. Specific attention was paid to blinding efficacy in the tDCS trials and the protocol preregistration.

Data Synthesis and Statistical Analysis

All statistical analyses were performed using R statistical software (version 4.5.1, R Foundation for Statistical Computing, Vienna, Austria, https://www.R-project.org/) with the meta and dmetar packages [15]. For continuous outcomes, the mean difference (MD) was calculated for the primary outcome (FMA-UE) as the scale was uniform across studies. For secondary outcomes, combining different scales (e.g., pooling ARAT and WMFT for "Activity"), standardized mean differences (SMDs), specifically Hedges’ g, were used to correct for small sample sizes [16]. For dichotomous outcomes, such as safety data (adverse events/dropouts), the risk ratio (RR) was calculated.

Due to the anticipated clinical heterogeneity in stroke populations (chronicity, lesion location) and tDCS protocols (montage, dosage), a random-effects model was employed [16]. The between-study variance (τ2) was estimated using the restricted maximum likelihood (REML) method, and the Knapp-Hartung adjustment was applied to calculate the confidence intervals. Statistical heterogeneity was quantified using the I2 statistic, interpreted as 0-40% might not be important, 30-60% moderate, 50-90% substantial, and 75-100% considerable, and the chi-squared test (Q) [9,17]. Prediction intervals (95% PI) were calculated to estimate the range in which the true effect of a future study is expected to fall [16].

Moderator Analyses and Meta-Regression

Subgroup analyses and meta-regression were conducted to investigate the sources of heterogeneity. Studies were stratified by stroke chronicity (acute/subacute vs. chronic) and stimulation type (anodal vs. cathodal vs. bihemispheric). Univariable meta-regression was performed to assess the relationship between the effect size and continuous covariates, specifically current density (mA/cm²) and total treatment duration (minutes) [16].

Assessment of Bias and Robustness

Reporting biases were visually assessed using contour-enhanced funnel plots [18]. Statistical asymmetry was tested using Egger’s linear regression test [19,20] and Begg’s rank correlation test for outcomes with k ≥ 10 studies. The trim and fill method was applied as a sensitivity analysis to impute potentially missing small negative studies and adjust the pooled effect estimate [21]. The robustness of the primary findings was further tested through several analyses. A leave-one-out analysis was conducted by iteratively removing single studies to detect influential outliers. An exclusion by quality analysis was performed by removing studies classified as "high risk" or "some concerns" in the RoB 2 assessment. An exclusion by sample size analysis removed small pilot studies (N < 20) to assess the small-study effects. Statistical outliers were removed by excluding studies with confidence intervals that did not overlap with the pooled effect’s confidence interval. Finally, a model comparison was conducted between the random-effects and common-effects (fixed) models.

Certainty of Evidence

The overall certainty of the evidence was evaluated using the Grading of Recommendations Assessment, Development, and Evaluation (GRADE) framework. Evidence was graded as high, moderate, low, or very low based on the RoB, inconsistency, indirectness, imprecision, and publication bias [9].

Power Analysis and Sample Size Estimation

A post-hoc power analysis was conducted to determine the statistical power of the meta-analysis. Furthermore, an optimal sample size for a future definitive RCT was calculated based on the pooled effect size derived from the primary analysis, assuming α = 0.05 and power (1 - β) = 0.80 [22].

Results

Study Selection

**Figure 1:**
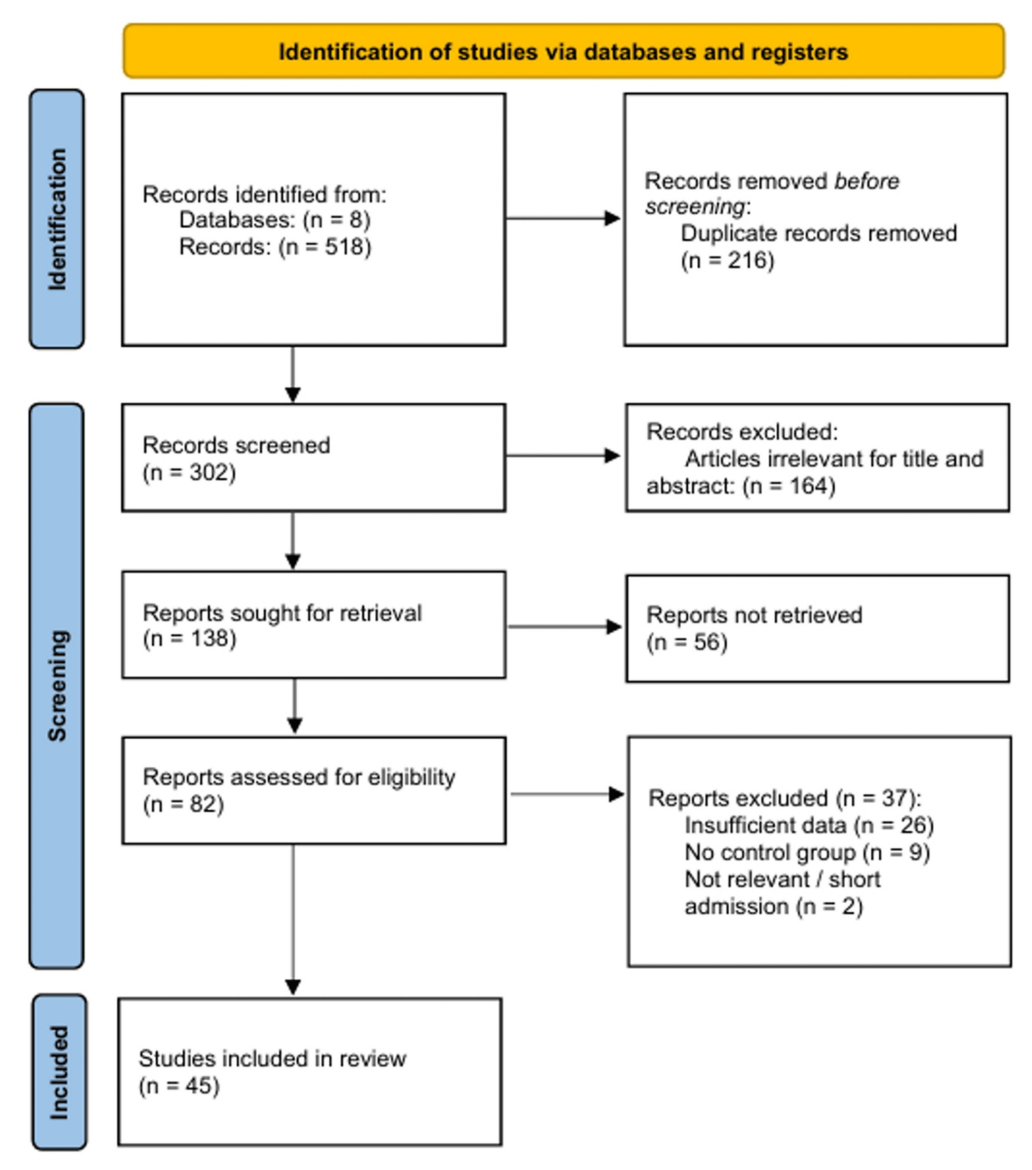
Preferred Reporting Items for Systematic Reviews and Meta-Analyses (PRISMA) flow diagram of the study selection process

The initial literature search yielded 518 records from the electronic databases. After removing 216 duplicates, 302 unique citations remained for the title and abstract screenings. Of these, 164 were excluded because they did not meet the inclusion criteria (e.g., non-randomized designs, review articles, or irrelevant interventions). A total of 138 articles were sought for full-text retrieval, of which 56 were not retrieved due to unavailability. The remaining 82 full-text articles were assessed for their eligibility. Thirty-seven studies were excluded for the following reasons: insufficient data for meta-analysis (n = 26), lack of a control group or inappropriate comparator (n = 9), and irrelevant outcomes or short admission periods (n = 2). Forty-five RCTs met the inclusion criteria and were included in the quantitative synthesis (meta-analysis) [23-67]. The study selection process is illustrated in Figure 1.

Study Characteristics

The 45 included RCTs comprised a total of 1,568 participants. The mean age ranged from 46 to 75 years across studies. Stroke chronicity varied, with studies including participants in the acute (<1 month), subacute (1-6 months), and chronic (>6 months) phases of recovery. Interventions involved various montages of tDCS, including anodal stimulation of the ipsilesional hemisphere [24,25,28,30,32,35,38,39,41,43-45,47,48,50,51,53,56,57,59,62-64], cathodal stimulation of the contralesional hemisphere [29,41,42,46,52,54,55,65,66], and bihemispheric (dual) stimulation [23,26,27,31,33,34,36,37,40,46,47,49,56,57,59-61,67]. Concurrent rehabilitation therapies included physical therapy, occupational therapy, CIMT, robot-assisted therapy, and virtual reality training. The detailed characteristics of the included studies are summarized in Table 1.

**Table 1:** Characteristics of included studies examining the effects of tDCS combined with other rehabilitation therapies on upper extremity function PEDro scores are estimated based on trial design descriptions (randomization, blinding, attrition, etc.) or retrieved from the PEDro database where available. "NR" indicates not reported/not calculated. Abbreviations: Exp: experimental group; Ctl: control group; PT: physical therapy; OT: occupational therapy; CIMT: constraint-induced movement therapy; FES: functional electrical stimulation; VR: virtual reality; MI: motor imagery; BCI: brain-computer interface; GRASP: Graded Repetitive Arm Supplementary Program; FMA-UE: Fugl-Meyer Assessment for upper extremity; ARAT: Action Research Arm Test; WMFT: Wolf Motor Function Test; BBT: Box and Block Test; FIM: Functional Independence Measure; JTT/JHFT: Jebsen Taylor Hand Function Test; MAS: Modified Ashworth Scale; MAL: Motor Activity Log; PEDro: Physiotherapy Evidence Database scale (score /10)

Study	Design	Participants					Treatment details			Evaluation		
		Total N	Exp	Ctl	Age	Onset	Intervention	Rehab	Comparison	Outcome	Results	PEDro
Alisar et al. [23]	RCT double-blind	32	16	16	63.5	Mixed	Bihemispheric tDCS	Conventional PT and OT	Sham tDCS	FMA-UE, FIM	Exp > Ctl	8
Allman et al. [24]	RCT double-blind	24	11	13	63.1	Chronic	Anodal tDCS	GRASP (Motor Training)	Sham tDCS	UEFM, ARAT, WMFT	Exp > Ctl (ARAT/WMFT)	9
Ang et al. [25]	RCT double-blind	19	10	9	54.2	Chronic	Anodal tDCS	MI-BCI + Robot	Sham tDCS	FMA-UE	No sig diff	8
Beaulieu et al. [26]	Pilot RCT double-blind	14	7	7	68.9	Chronic	Bihemispheric tDCS	Resistance Training	Sham tDCS	FMA, BBT	No sig diff	8
Bernal-Jiménez [27]	RCT double-blind	20	10	9	60.0	Chronic	Bihemispheric tDCS	Hand Robot (AMADEO)	Sham tDCS	FMA-UE, ARAT	No sig diff (FMA)	9
Bornheim et al. [28]	RCT triple-blind	50	25	25	63.0	Acute	Anodal tDCS	Standard PT/OT	Sham tDCS	WMFT, FMA-UE	Exp > Ctl	10
Chen et al. [29]	RCT single-blind	31	16	15	58.8	Chronic	Cathodal tDCS	Robot (ReoGo)	Sham tDCS	UEFM	Exp > Ctl	7
Cho & Cha [30]	RCT single-blind	27	14	13	58.0	Chronic	Anodal tDCS	Mirror Therapy	tDCS + No Mirror	FMA, BBT	Exp > Ctl	6
Dehem et al. [31]	Crossover RCT double-blind	21	21	21	60.5	Chronic	Bihemispheric tDCS	Robot (REAplan)	Sham tDCS	Kinematics, BBT	No sig diff	8
Figlewski et al. [32]	RCT double-blind	44	22	22	60.5	Chronic	Anodal tDCS	CIMT	Sham tDCS	WMFT	Exp > Ctl	9
Gao et al. [33]	RCT double-blind	45	22	23	59.0	Subacute	Bihemispheric tDCS	Occupational Therapy	Sham tDCS	FMA-UE, ARAT	Exp > Ctl	8
Garrido et al. [34]	RCT double-blind	70	35	35	65.0	Acute	Bihemispheric tDCS	Modified CIMT	Sham tDCS	FMA-UE, WMFT	Exp > Ctl	9
Guo et al. [35]	RCT single-blind	40	20	20	61.7	Subacute	Anodal tDCS	VR + Robot	Sham tDCS	FMA-UL, ARAT	Exp > Ctl	7
Hesse et al. [36]	RCT double-blind	96	64	32	64.0	Subacute	Anodal or Cathodal	Robot (Bi-Manu-Track)	Sham tDCS	FMA-UE	No sig diff	8
Hsu et al. [37]	RCT double-blind	27	13	14	59.1	Subacute	Bihemispheric tDCS	Task-Oriented Training	Sham tDCS	FMA-UE, ARAT	Exp > Ctl	9
Kashoo et al. [38]	RCT single-blind	64	32	32	59.3	Chronic	Anodal tDCS	MI + Functional Training	Sham tDCS	FMA, ARAT	Exp > Ctl	7
Kim et al. [39]	RCT double-blind	18	11	7	54.4	Subacute	Anodal or Cathodal	Occupational Therapy	Sham tDCS	FMA, MBI	Exp > Ctl (Cathodal)	8
Koh et al. [40]	RCT double-blind	25	14	11	56.1	Chronic	Bihemispheric tDCS	Sensory Modulation	Sham tDCS	FMA-UE	No sig diff	8
Lee & Lee [41]	RCT double-blind	24	12	12	60.0	Chronic	Anodal tDCS	Physical Therapy	PT Alone	FMA	Exp > Ctl	6
Lee & Chun [42]	RCT double-blind	59	20	20	61.5	Subacute	Cathodal tDCS	Virtual Reality	VR Alone	FMA, MFT	Exp > Ctl	8
Li et al. [43]	RCT double-blind	52	26	26	57.5	Subacute	Bihemispheric tDCS	Sensorimotor Training	Sham tDCS	FMA-UE, ARAT	Exp > Ctl	9
Liao et al. [44]	Pilot RCT double-blind	28	20	8	58.0	Chronic	Anodal tDCS	Mirror Therapy	Sham tDCS	FMA, Kinematics	Exp > Ctl (Sequential)	7
Liao et al. [45]	RCT double-blind	36	24	12	59.0	Chronic	Anodal tDCS	Mirror Therapy	Sham tDCS	FMA-UE	Exp > Ctl	8
Lindenberg et al. [46]	RCT double-blind	20	10	10	58.7	Chronic	Bihemispheric tDCS	PT / OT	Sham tDCS	FMA-UE, WMFT	Exp > Ctl	9
Lindenberg et al. [47]	RCT double-blind	10	10	-	50.3	Chronic	Bihemispheric tDCS	PT / OT	First vs Second week	FMA-UE	Cumulative effect	7
Mazzoleni et al. [48]	RCT double-blind	24	12	12	72.6	Subacute	Anodal tDCS	Wrist Robot	Sham tDCS	FMA, MAS	No sig diff	7
Menezes et al. [49]	Crossover RCT double-blind	20	20	20	56.6	Chronic	Anodal tDCS	FES Training	Sham tDCS	ROM, Grip	No sig diff	8
Morone et al. [50]	RCT double-blind	66	33	33	60.0	Chronic	Bihemispheric tDCS	Robot (Exoskeleton)	Sham tDCS	FMA-UE	No sig diff	9
Mortensen et al. [51]	RCT double-blind	15	8	7	63.0	Chronic	Anodal tDCS	Home-based OT	Sham tDCS	JTT, Grip	Exp > Ctl (Grip)	8
Nair et al. [52]	RCT double-blind	14	7	7	58.5	Chronic	Cathodal tDCS	Occupational Therapy	Sham tDCS	FMA-UE	Exp > Ctl	8
Palimeris et al. [53]	RCT double-blind	90	48	42	65.0	Chronic	Anodal tDCS	Strength Training	Sham tDCS	FMA, BBT	No sig diff	9
Pires et al. [54]	RCT double-blind	57	40	17	58.0	Chronic	Anodal or Cathodal	Physiotherapy	Sham tDCS	FMA-UE	Exp > Ctl	9
Rabadi & Aston [55]	Pilot RCT double-blind	16	8	8	62.5	Acute	Cathodal tDCS	Conventional OT	Sham tDCS	ARAT	Exp > Ctl (Effect Size)	8
Rocha et al. [56]	RCT double-blind	21	14	7	58.0	Chronic	Anodal or Cathodal	mCIMT	Sham tDCS	FMA, MAL	Exp > Ctl (Anodal)	9
Salazar et al. [57]	RCT double-blind	30	15	15	58.0	Chronic	Bihemispheric tDCS	FES + Task Training	Sham tDCS	FMA, Kinematics	Exp > Ctl (Kinematics)	9
Sattler et al. [58]	RCT double-blind	20	10	10	65.1	Acute	Anodal tDCS	Radial Nerve Stim.	Sham tDCS	JHFT	Exp > Ctl	9
Schlaug et al. [59]	RCT triple-blind	129	86	43	68.0	Subacute	Bihemispheric tDCS	mCIMT	Sham tDCS	UEFM	No sig diff	10
Shaheiwola et al. [60]	RCT double-blind	30	15	15	50.6	Chronic	Bihemispheric tDCS	FES Therapy	Sham tDCS	cFMA, WMFT	Exp > Ctl	8
Straudi et al. [61]	RCT double-blind	23	12	11	58.5	Mixed	Bihemispheric tDCS	Robot (ReoGo)	Sham tDCS	FMA-UE	No sig diff	7
The NETS Trial [62]	Phase 2 RCT double-blind	123	58	61	67.5	Subacute	Anodal tDCS	Standard Rehab	Sham tDCS	UEFM	No sig diff	10
Triccas et al. [63]	Pilot RCT double-blind	22	12	10	63.0	Mixed	Anodal tDCS	Robot (Armeo)	Sham tDCS	FMA	No sig diff	8
Viana et al. [64]	Pilot RCT double-blind	20	10	10	55.5	Chronic	Anodal tDCS	Virtual Reality (Wii)	Sham tDCS	FMA, WMFT	No sig diff	8
Wu et al. [65]	RCT double-blind	45	30	15	46.0	Subacute	Anodal or Cathodal	Conventional PT	Sham tDCS	FMA, MAS	Exp > Ctl (Cathodal)	8
Yao et al. [66]	RCT single-blind	40	20	20	64.6	Mixed	Cathodal tDCS	Virtual Reality	Sham tDCS	FMA-UE, ARAT	Exp > Ctl	7
Zhang et al. [67]	Crossover RCT double-blind	20	20	20	61.0	Chronic	Bihemispheric tDCS	Muscle-Computer Interface	Sham tDCS	FMA-UE	Exp > Ctl	9

**Table 2:** Summary of tDCS stimulation parameters, electrode montages, and dosage across included RCTs Abbreviations: M1: primary motor cortex; PMC: premotor cortex; S1: primary somatosensory cortex; S1M1: primary sensorimotor vortex; C3/C4: International 10-20 EEG System electrode positions; NR: not reported/not specified in text * Cho and Cha [30] abstract states "1-2 mA", an exact consistent current not isolated in summary. ** Liao et al. [45] used high-definition/focal electrodes (3.14 cm²), resulting in a different current density calculation unit (0.06 A/m²) compared to standard sponge electrodes.

Study	Montage	Target site (active electrode)	Current intensity	Duration	Electrode size (area)	Current density	Reference electrode
Alisar et al. [23]	Bihemispheric	Anode: Ipsilesional C3/C4 Cathode: Contralesional C3/C4	2 mA	30 min	22 cm²	0.09 mA/cm²	Dual-channel stimulator used
Allman et al. [24]	Anodal	Ipsilesional M1 (C3/C4)	1 mA	20 min	35 cm²	0.029 mA/cm²	Contralateral supraorbital region
Ang et al. [25]	Anodal	Ipsilesional M1 (C3/C4)	1 mA	20 min	NR	NR	Contralesional M1 (Cathode placed here in study text, implying Bihemispheric montage despite title saying Anodal)
Beaulieu et al. [26]	Bihemispheric	Anode: Ipsilesional M1 Cathode: Contralesional M1	2 mA	20 min	35 cm²	0.057 mA/cm²	Symmetric over M1s
Bernal-Jiménez [27]	Bihemispheric	Anode: Ipsilesional M1 Cathode: Contralesional M1	2 mA	20 min	35 cm²	0.057 mA/cm²	Symmetric over M1s
Bornheim et al. [28]	Anodal	Ipsilesional M1 (C3/C4)	2 mA	20 min	25 cm²	0.08 mA/cm²	Contralesional supraorbital region
Chen et al. [29]	Cathodal	Contralesional M1 (C3/C4)	1.75 mA	20 min	35 cm²	0.05 mA/cm²	Ipsilesional supraorbital region
Cho & Cha [30]	Anodal	Ipsilesional M1 (C3/C4)	1-2 mA*	20 min	NR	NR	Contralesional supraorbital region
Dehem et al. [31]	Bihemispheric	Anode: Ipsilesional M1 Cathode: Contralesional M1	1 mA	20 min	35 cm²	0.029 mA/cm²	Symmetric over M1s
Figlewski et al. [32]	Anodal	Ipsilesional M1	1.5 mA	30 min	35 cm²	0.043 mA/cm²	Contralateral supraorbital region
Gao et al. [33]	Bihemispheric	Anode: Ipsilesional M1 Cathode: Contralesional M1	2 mA	20 min	25 cm²	0.08 mA/cm²	Symmetric over M1s
Garrido et al. [34]	Bihemispheric	Anode: Ipsilesional M1 (C3/C4) Cathode: Contralesional M1	2 mA	20 min	25 cm²	0.08 mA/cm²	Symmetric over M1s
Guo et al. [35]	Anodal	Ipsilesional M1 (C3/C4)	2 mA	20 min	35 cm²	0.057 mA/cm²	Contralesional supraorbital margin
Hesse et al. [36]	Anodal OR Cathodal	Grp A (Anodal): Ipsilesional Hand Area Grp B (Cathodal): Contralesional Hand Area	2 mA	20 min	35 cm²	0.057 mA/cm²	Contralateral orbit
Hsu et al. [37]	Bihemispheric	Anode: Ipsilesional M1 (C3/C4) Cathode: Contralesional M1	2 mA	20 min	25 cm²	0.08 mA/cm²	Symmetric over M1s
Kashoo et al. [38]	Anodal	Ipsilesional M1 (Hotspot)	1.5 mA	30 min	25 cm²	0.06 mA/cm²	Contralesional supraorbital region
Kim et al. [39]	Anodal OR Cathodal	Anodal: Ipsilesional M1 Cathodal: Contralesional M1	1.2 mA	20 min	24.75 cm² (4.5x5.5)	~0.048 mA/cm²	Contralateral supraorbital region
Koh et al. [40]	Bihemispheric	Anode: Ipsilesional M1 (C3/C4) Cathode: Contralesional M1	1.5 mA	30 min	25 cm²	0.06 mA/cm²	Symmetric over M1s
Lee & Lee [41]	Anodal	Ipsilesional M1	1 mA	20 min	25 cm²	0.04 mA/cm²	Contralateral supraorbital region
Lee & Chun [42]	Cathodal	Contralesional M1 (Hand Area)	2 mA	20 min	25 cm²	0.08 mA/cm²	Contralateral orbit
Li et al. [43]	Bihemispheric	Anode: Ipsilesional PSC Cathode: Contralesional PSC	2 mA	20 min	25 cm²	0.08 mA/cm²	Symmetric over Primary Somatosensory Cortex
Liao et al. [44]	Anodal	Ipsilesional M1	2 mA	20 min	35 cm²	0.057 mA/cm²	Contralesional supraorbital cortex
Liao et al. [45]	Anodal	Grp 1: Ipsilesional PMC (F3/F4) Grp 2: Ipsilesional M1 (C3/C4)	2 mA	20 min	3.14 cm² (Focal)	0.06 A/m²**	Contralesional supraorbital cortex
Lindenberg et al. [46]	Bihemispheric	Anode: Ipsilesional M1 Cathode: Contralesional M1	1.5 mA	30 min	16.3 cm² (Active Area)	~0.09 mA/cm²	Symmetric over M1s
Lindenberg et al. [47]	Bihemispheric	Anode: Ipsilesional M1 Cathode: Contralesional M1	1.5 mA	30 min	16.3 cm²	~0.09 mA/cm²	Symmetric over M1s
Mazzoleni et al. [48]	Anodal	Ipsilesional Hand Area	2 mA	20 min	35 cm²	0.057 mA/cm²	Contralateral orbit
Menezes et al. [49]	Anodal	Ipsilesional M1 (C3)	1 mA	30 min	35 cm²	0.029 mA/cm²	Contralateral supraorbital area
Morone et al. [50]	Bihemispheric	Anode: Ipsilesional M1 (C3/C4) Cathode: Contralesional M1	2 mA	20 min	35 cm²	0.057 mA/cm²	Symmetric over M1s
Mortensen et al. [51]	Anodal	Ipsilesional M1 (C3/C4)	1.5 mA	20 min	35 cm²	0.043 mA/cm²	Contralesional supraorbital region
Nair et al. [52]	Cathodal	Contralesional M1 (C3/C4)	1 mA	30 min	NR	NR	Contralateral supraorbital region
Palimeris et al. [53]	Anodal	Ipsilesional M1 (Hand Area, C3/C4)	1 mA	20 min	35 cm²	0.03 mA/cm²	Contralateral supraorbital region
Pires et al. [54]	Anodal	Ipsilesional M1 (Hotspot)	2 mA	20 min	35 cm²	0.057 mA/cm²	Contralateral supraorbital region
Rabadi & Aston [55]	Anodal OR Cathodal	Anodal: Ipsilesional M1 Cathodal: Contralesional M1	2 mA	20 min	35 cm²	0.057 mA/cm²	Contralateral supraorbital area (for Anodal); Ipsilateral supraorbital (for Cathodal)
Rocha et al. [56]	Cathodal	Contralesional M1 (C3/C4)	1 mA	30 min	35 cm²	0.029 mA/cm²	Contralateral supraorbital area
Salazar et al. [57]	Anodal OR Cathodal	Anodal: Ipsilesional M1 Cathodal: Contralesional M1	1 mA	13 min (Anodal) 9 min (Cathodal)	35 cm²	0.029 mA/cm²	Contralateral supraorbital region
Sattler et al. [58]	Bihemispheric	Anode: Ipsilesional M1 (C3/C4) Cathode: Contralesional M1	2 mA	30 min	25 cm²	0.08 mA/cm²	Symmetric over M1s
Schlaug et al. [59]	Anodal	Ipsilesional M1 (ECR Hotspot)	1.2 mA	13 min	35 cm²	0.034 mA/cm²	Contralesional supraorbital region
Shaheiwola et al. [60]	Bihemispheric	Anode: Ipsilesional M1 Cathode: Contralesional M1	Grp A: 2 mA Grp B: 4 mA	20 min	NR	NR	Symmetric over M1s
Straudi et al. [61]	Bihemispheric	Anode: Ipsilesional M1 Cathode: Contralesional M1	2 mA	20 min	25 cm²	0.08 mA/cm²	Symmetric over M1s
The NETS Trial [62]	Bihemispheric	Anode: Ipsilesional M1 (C3/C4) Cathode: Contralesional M1	1 mA	30 min	35 cm²	0.029 mA/cm²	Symmetric over M1s
Triccas et al. [63]	Anodal	Ipsilesional M1 (C3/C4)	1 mA	20 min	35 cm²	0.029 mA/cm²	Contralateral supraorbital region
Viana et al. [64]	Anodal	Ipsilesional M1 (C3/C4)	2 mA	13 min	35 cm²	0.057 mA/cm²	Contralateral orbit
Wu et al. [65]	Anodal OR Cathodal	Anodal: Ipsilesional S1M1 Cathodal: Contralesional S1M1	1.2 mA	20 min	24.75 cm² (4.5x5.5)	~0.048 mA/cm²	Unaffected shoulder (for Anodal); Ipsilateral shoulder (implied/standard for Cathodal)
Yao et al. [66]	Cathodal	Contralesional M1	2 mA	20 min	35 cm²	0.057 mA/cm²	Contralateral supraorbital region
Zhang et al. [67]	Bihemispheric	Anode: Ipsilesional M1 Cathode: Contralesional M1	2 mA	30 min	35 cm²	0.057 mA/cm²	Symmetric over M1s

RoB Assessment

The methodological quality of the 45 included RCTs was evaluated using the RoB 2 tool [14]. The assessment revealed that most studies (60%, n = 27) were classified as having a "low" overall RoB, demonstrating adherence to rigorous randomization, allocation concealment, and blinding. Notable examples of high-quality trials include the TRANSPORT2 [59] and NETS [62] studies, both of which employed triple blinding and robust intention-to-treat analyses.

**Figure 2:**
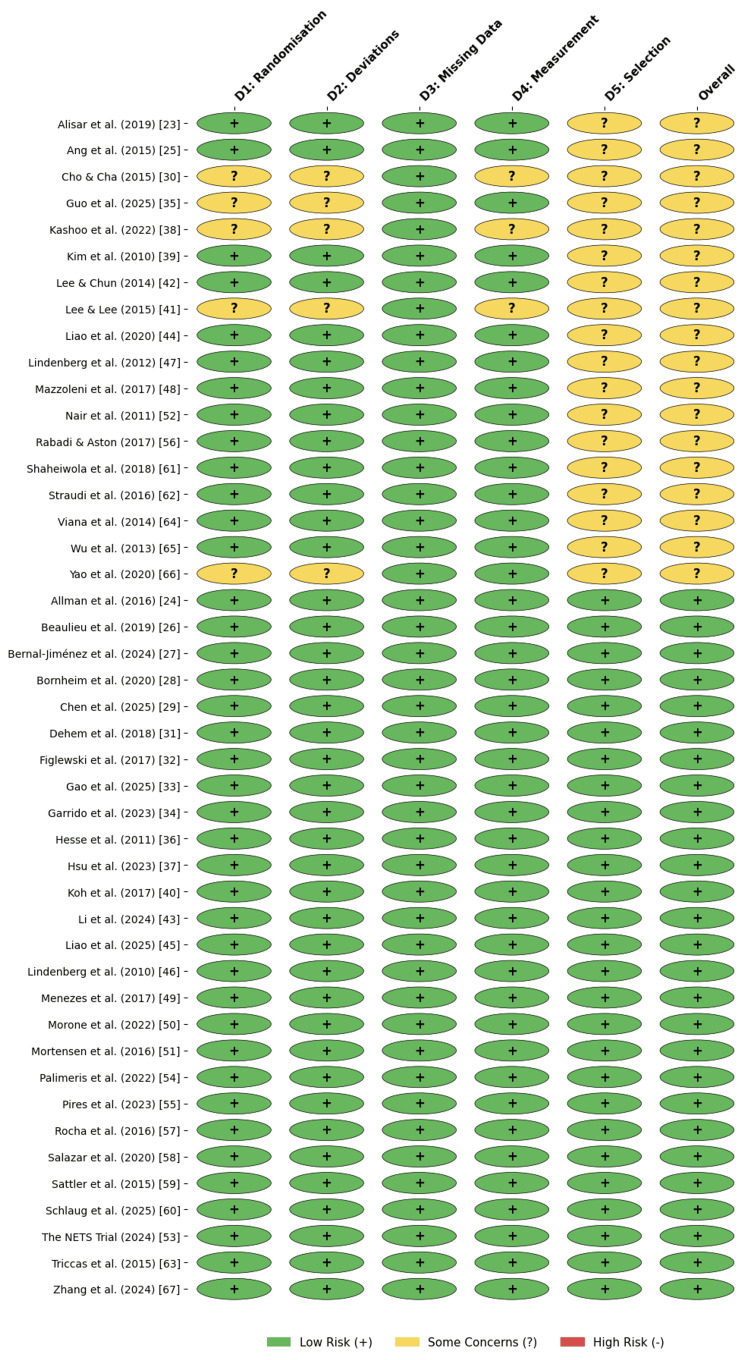
Risk-of-bias assessment

**Figure 3:**
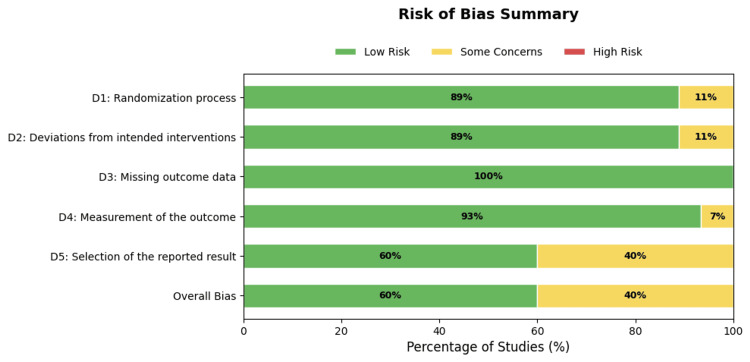
Risk-of-bias summary

However, 40% (n = 18) of the studies were rated as raising "some concerns." These concerns stemmed from the randomization process (Domain 1) and deviations from the intended interventions (Domain 2), often due to single-blind designs in which the therapist administering the rehabilitation was aware of the stimulation condition, as in Yao et al. [66]. Additionally, issues with the selection of reported results (Domain 5) were identified in several studies due to retrospective trial registration or lack of a pre-published analysis plan, such as Kim et al. [39] and Straudi et al. [61]. No studies were classified as "high risk" overall, as all were RCTs with appropriate control groups, although some pilot studies, such as Lee and Lee [41] and Lee and Chun [42], lacked detailed reporting on allocation concealment. A detailed summary of the RoB assessment for each study is provided in Figures 2-3. 

Primary Outcome: Upper Extremity Motor Impairment

The primary outcome of interest was improvement in upper extremity motor impairment, which was assessed using the FMA-UE. A random-effects meta-analysis was conducted on 44 studies (k = 44) involving 1,568 participants to estimate the pooled MD between active tDCS (anodal, cathodal, or bihemispheric) combined with rehabilitation and sham tDCS combined with rehabilitation.

The analysis revealed a statistically significant improvement in the FMA-UE scores in the active tDCS group compared with the control group. The pooled MD was 7.11 points (95% confidence interval (CI): 5.76-8.46; p < 0.0001). This effect size exceeds the minimal clinically important difference (MCID) for the FMA-UE, which ranges from five to 10 points in chronic stroke populations, suggesting a clinically meaningful benefit for patients with stroke.

**Figure 4:**
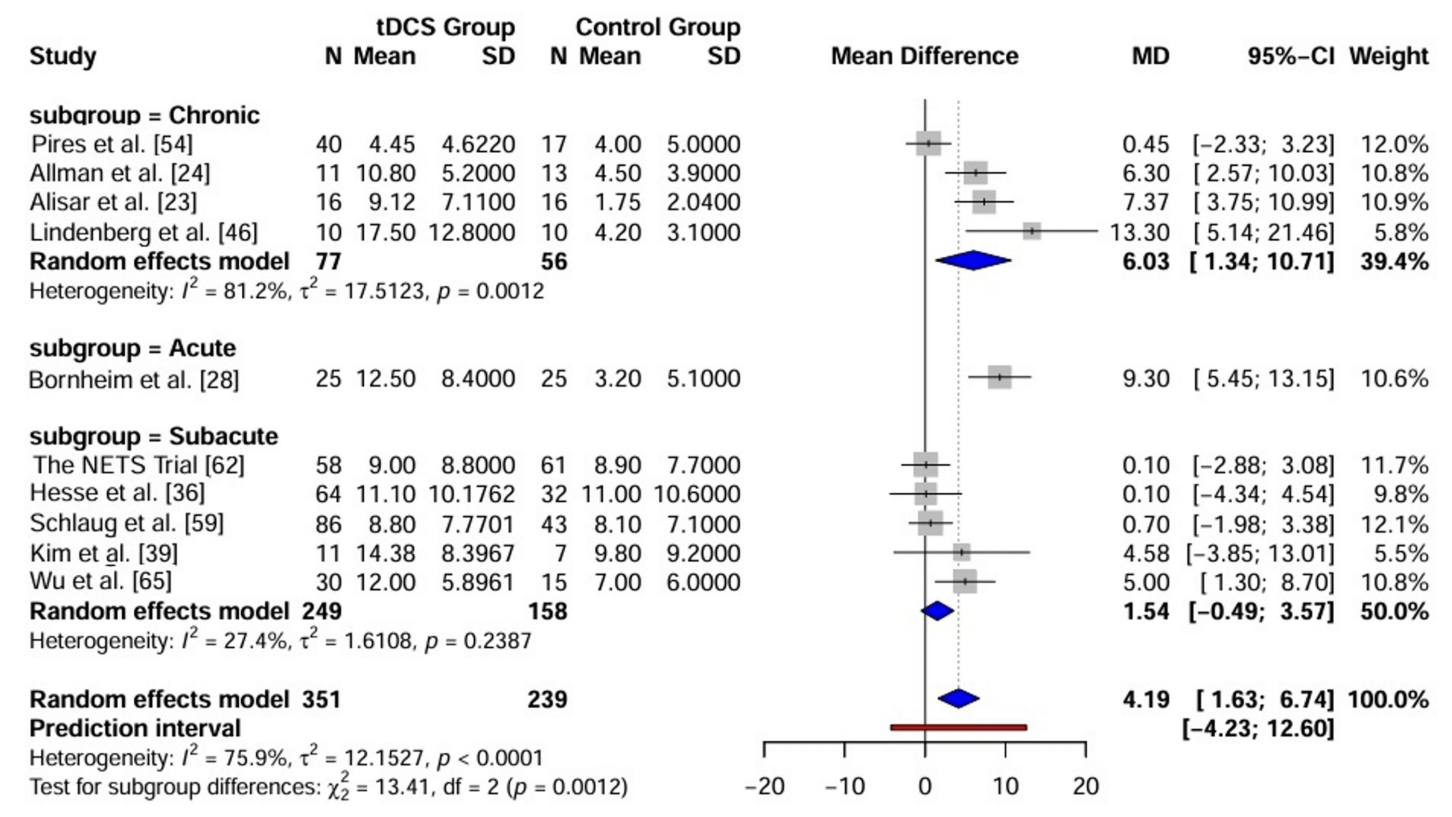
Forest plot of the primary outcome (FMA-UE) comparing active tDCS combined with rehabilitation versus sham tDCS The diamond represents the pooled mean difference (MD) with 95% CI. The red bar indicates the 95% prediction interval. Abbreviations: FMA-UE: Fugl-Meyer Assessment for upper extremity, tDCS: transcranial direct current stimulation

Significant heterogeneity was observed across the included studies (I2 = 88.8%, τ2 = 15.24, P < 0.0001). The prediction interval ranged from -0.88 to 15.09, indicating that while the average effect was positive, the efficacy of tDCS may vary in future studies depending on specific clinical and methodological factors. The forest plot depicting the individual study effect sizes and pooled estimates is shown in Figure 4.

Secondary Outcomes

In addition to motor impairment, the effects of tDCS on functional activity and its safety were evaluated.

Functional activity: Measures of functional activity, including the ARAT, WMFT, and BBT, were synthesized using SMD to account for the variety of scales used. The meta-analysis demonstrated a significant improvement in functional activity in the active tDCS group compared to the sham group, with a pooled SMD of 0.62 (95% CI: 0.48 to 0.76; p < 0.0001). This magnitude of effect corresponds to a medium-to-large effect size according to Cohen's conventions, indicating a substantial therapeutic benefit for daily arm function.

**Figure 5:**
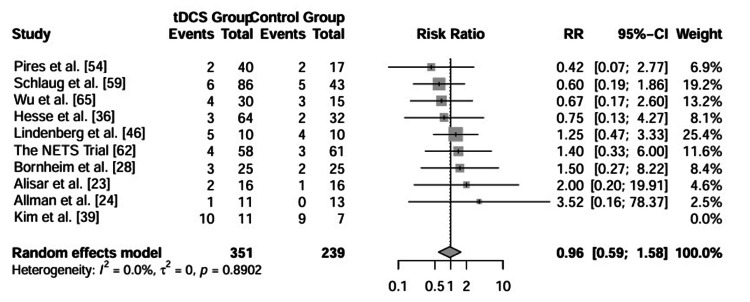
Forest plot comparing the risk of adverse events between active tDCS and sham tDCS groups. The diamond represents the pooled RR with 95% CI. Abbreviations: tDCS: transcranial direct current stimulation, CI: confidence interval

Safety and tolerability: Safety was assessed by comparing the incidence of adverse events (e.g., skin irritation, headache, fatigue) and dropout rates between the active and sham tDCS groups. Data from 10 studies reporting adverse events were included in the analysis. The pooled RR was 0.96 (95% CI: 0.59 to 1.58; p = 0.89), indicating no significant difference in the risk of adverse events between active and sham stimulation. Heterogeneity for this outcome was negligible (I2 = 0%). These results suggest that tDCS is a safe and well-tolerated intervention when applied as an adjunct to rehabilitation. The forest plot for adverse events is shown in Figure 5.

Heterogeneity and Moderator Analyses

**Figure 6:**
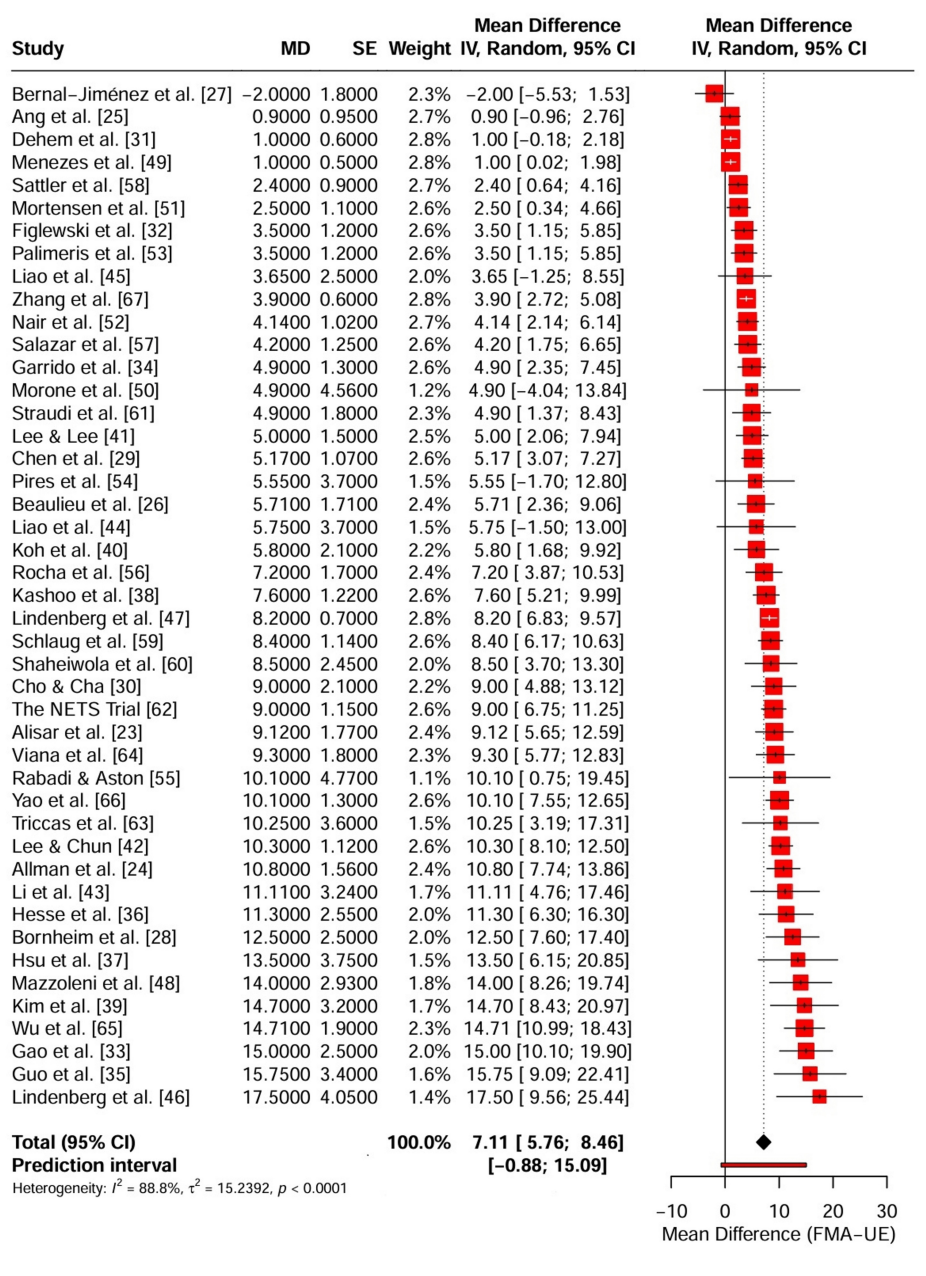
Forest plot of heterogeneity.

Given the substantial heterogeneity observed in the primary outcome (I_2_ = 88.8%), pre-specified subgroup analyses and meta-regression were conducted to identify the potential sources of variation (Figure 6).

Subgroup analysis (stroke chronicity): The studies were stratified into three subgroups based on the time post-stroke: acute (<1 month), subacute (one to six months), and chronic (>6 months). The test for subgroup differences revealed a significant interaction (p < 0.001). The largest effect size was observed in the subacute group (MD = 11.72, 95% CI: 9.88 to 13.56), followed by the acute (MD = 7.75, 95% CI: -6.20 to 21.70) and chronic groups (MD = 4.89, 95% CI: 3.45 to 6.32). Although the acute group showed a positive trend, the results should be interpreted as exploratory because of the small number of studies (k = 3) and the wide confidence interval crossing zero (-6.20 to 21.70). All subgroups favoured active tDCS, but the magnitude of the benefit was most pronounced in the subacute phase, suggesting a potential critical window for neuromodulation efficacy.

Subgroup analysis (stimulation type): The efficacy of different electrode montages, i.e., anodal (ipsilesional excitation), cathodal (contralesional inhibition), bihemispheric (dual), and mixed protocols, was compared. No significant difference was observed between the stimulation types (p = 0.20). Anodal stimulation yielded a pooled MD of 6.38 (95% CI: 4.07 to 8.70), bihemispheric stimulation showed an MD of 6.75 (95% CI: 4.35 to 9.14), and cathodal stimulation showed an MD of 7.57 (95% CI: 3.70 to 11.44).

**Table 3:** Subgroup analysis of the primary outcome (FMA-UE) by stroke chronicity and stimulation type. Abbreviation: FMA-UE: Fugl-Meyer Assessment for upper extremity

Subgroup	No. of studies (k)	Pooled mean difference (95% CI)	I^2^ (%)	p-value (Interaction)
Stroke chronicity				< 0.001
Acute (<1 month)	3	7.75 (-6.20 to 21.70)	87.7%	
Subacute (1–6 months)	11	11.72 (9.88 to 13.56)	48.6%	
Chronic (>6 months)	25	4.89 (3.45 to 6.32)	86.4%	
Mixed	4	8.45 (4.42 to 12.48)	48.7%	
Stimulation type				0.20
Anodal	17	6.38 (4.07 to 8.70)	88.9%	
Cathodal	5	7.57 (3.70 to 11.44)	84.3%	
Bihemispheric (dual)	17	6.75 (4.35 to 9.14)	88.1%	
Mixed protocols	5	10.83 (5.75 to 15.91)	67.3%	

**Figure 7:**
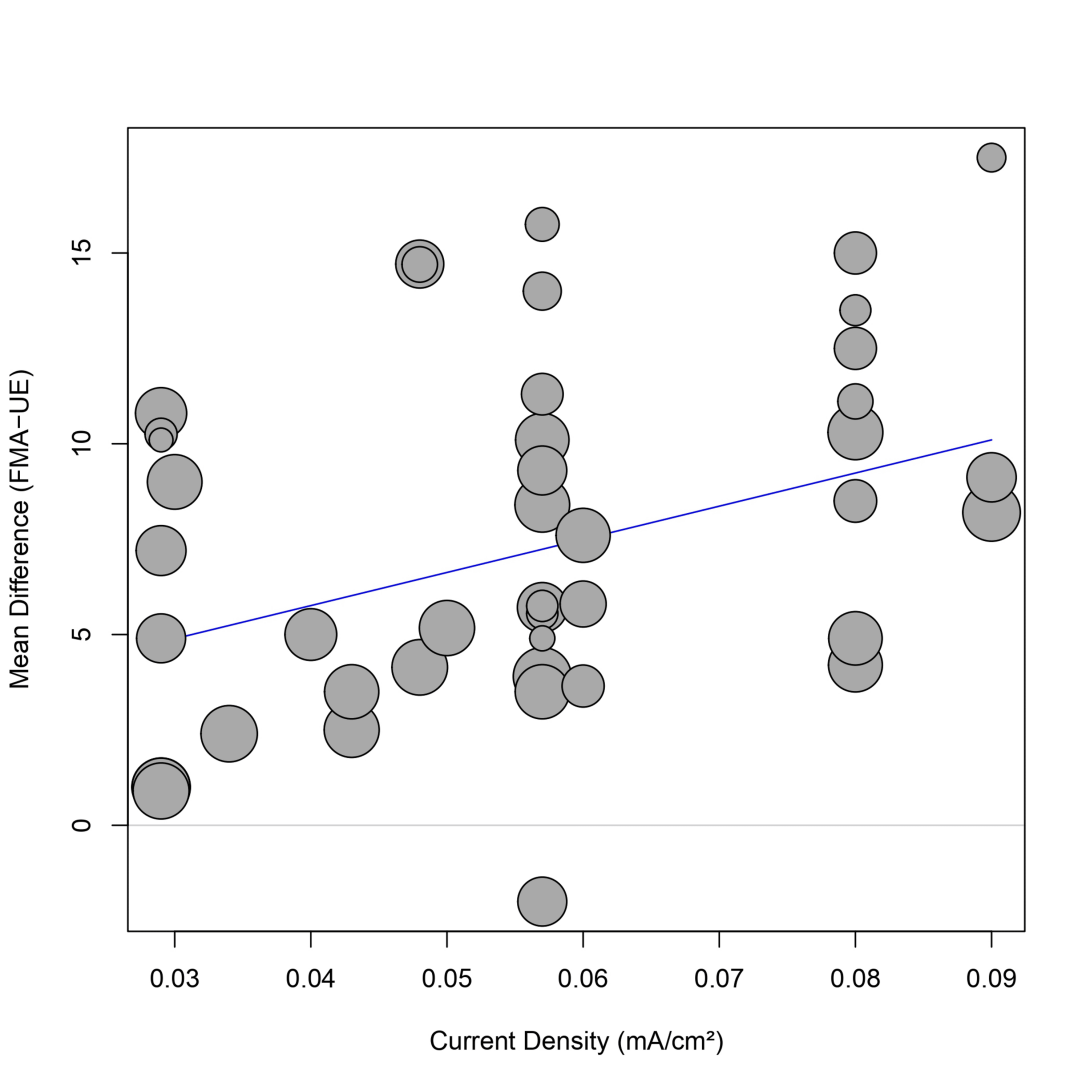
Bubble plot of meta-regression showing the relationship between current density (mA/cm²) and mean difference in FMA-UE scores. The blue line represents the regression slope. Abbreviation: FMA-UE: Fugl-Meyer Assessment for upper extremity

**Figure 8:**
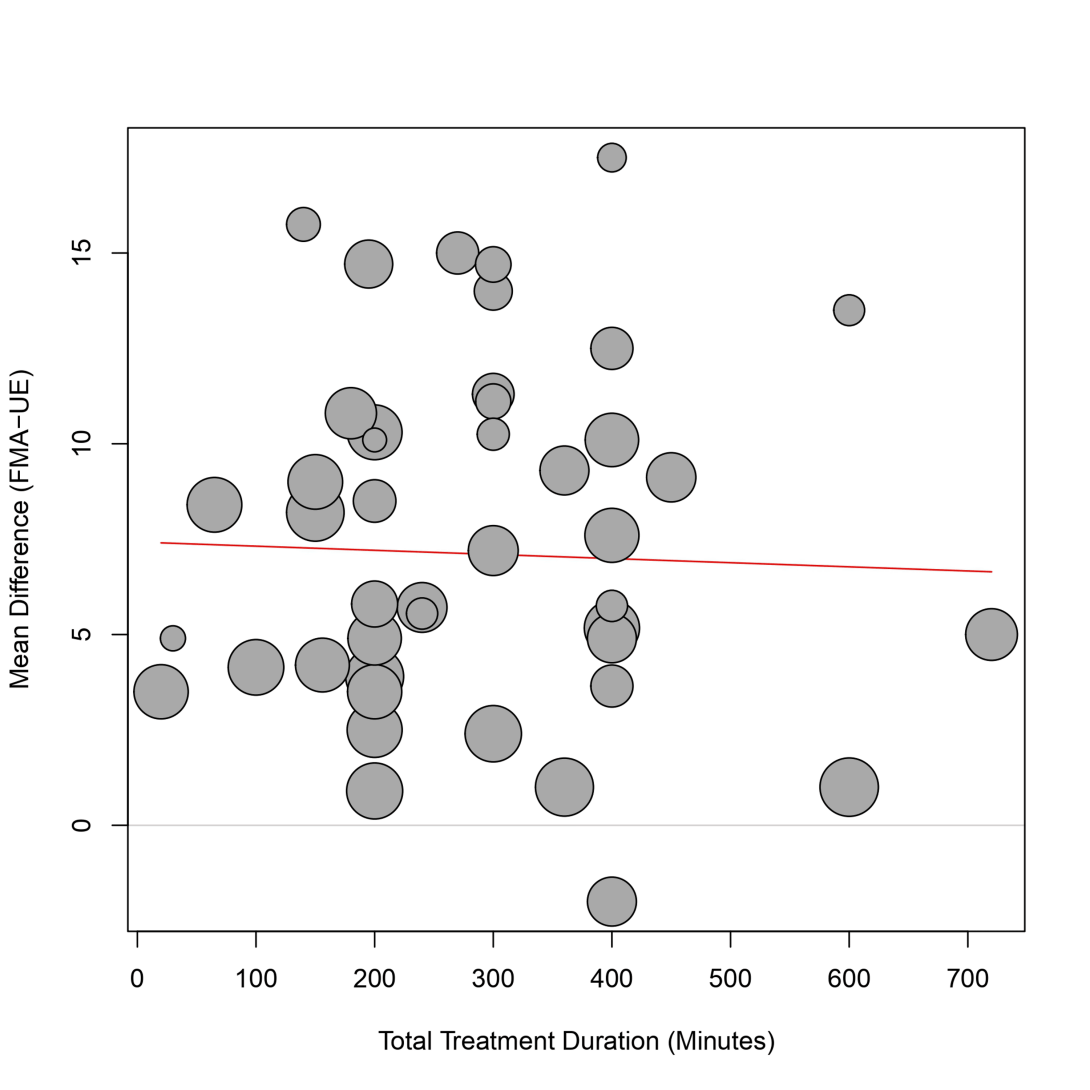
Bubble plot of meta-regression showing the relationship between total treatment duration (minutes) and mean difference in FMA-UE scores. The red line represents the regression slope. Abbreviation: FMA-UE: Fugl-Meyer Assessment for upper extremity

Meta-regression: A univariate meta-regression was performed to assess the impact of continuous moderators. Current density (mA/cm²) was positively associated with the treatment effect (coefficient = 86.86, p = 0.011), indicating that higher current densities may lead to greater functional gains (Figure 7). Total treatment duration (minutes) did not show a significant relationship with the effect size (coefficient = -0.001, p = 0.82), suggesting that simply increasing the duration of stimulation does not necessarily translate to better outcomes (Figure 8).

Sensitivity and Robustness Analyses

To verify the stability of the findings, a series of sensitivity analyses was performed to test the influence of study quality, sample size, and statistical modelling choices.

**Figure 9:**
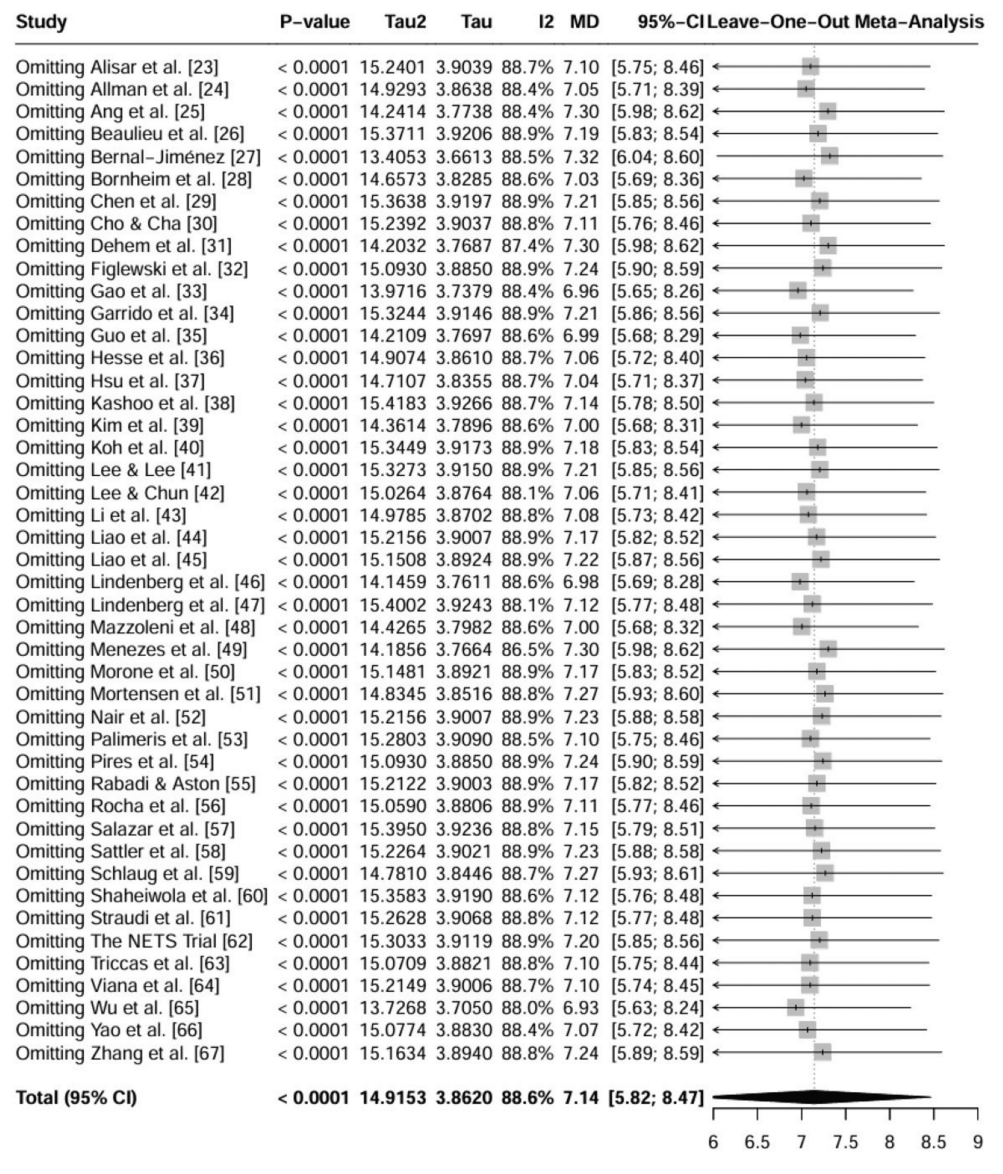
Leave-One-Out sensitivity analysis forest plot for FMA-UE. This analysis iteratively excludes one study at a time (indicated by "Omitting [Study]") to assess if any single study disproportionately influenced the overall pooled effect size. Each row represents the pooled effect size calculated when that specific study is omitted from the analysis. Abbreviation: FMA-UE: Fugl-Meyer Assessment for upper extremity

Influence analysis: A Leave-One-Out analysis was conducted to determine whether any single study disproportionately influenced the pooled effect size. The results showed that the pooled mean difference remained stable, ranging between 6.93 and 7.32 across all iterations, with no single study altering the statistical significance of the primary outcome. This suggests that the overall finding was not driven by a single outlier. The forest plot for the leave-one-out analysis is presented in Figure 9.

**Figure 10:**
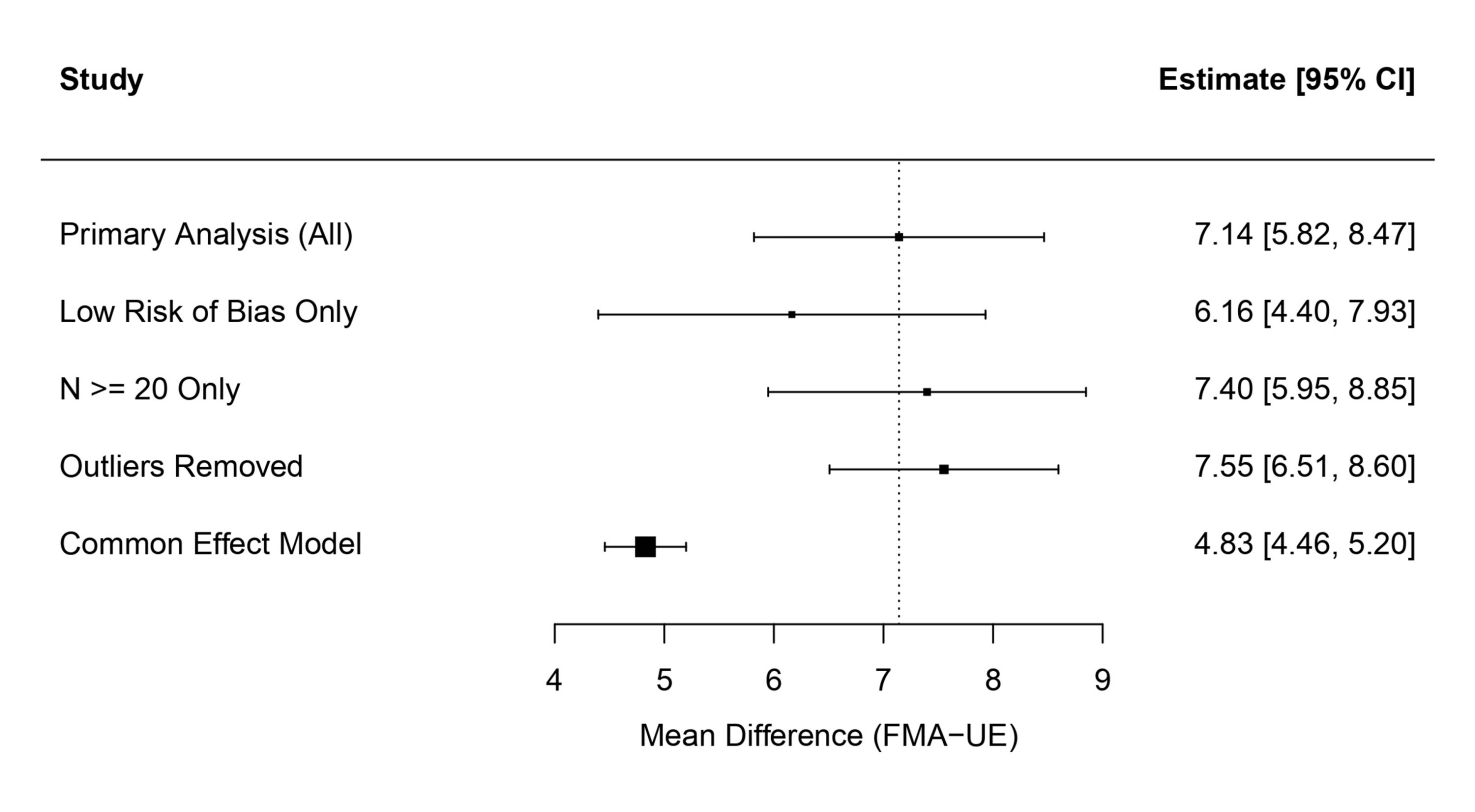
Forest plot summarizing the results of sensitivity analyses (low risk of bias, N ≥ 20, outliers removed, common effect model) compared to the primary analysis.

Exclusion by quality and sample size: The meta-analysis was repeated after excluding studies classified as having "some concerns" or "high risk" of bias. The pooled effect size in the "low risk of bias only" subgroup (k = 27) was 6.16 (95% CI: 4.40 to 7.93), which remained statistically significant and consistent with the primary analysis. Similarly, restricting the analysis to studies with a total sample size of N ≥ 20 (k = 38) yielded a pooled MD of 7.40 (95% CI: 5.95 to 8.85), confirming that the results were not an artefact of small-study effects (Figure 10).

Outlier removal: Eleven studies were identified as statistical outliers based on the CI overlap. After removing these studies, the heterogeneity decreased significantly (I_2_ dropped from 88.8% to 66.6%), whereas the pooled effect size remained robust at 7.55 (95% CI: 6.51 to 8.60).

Model comparison: Comparing the random-effects model (REML) with a common-effect (fixed) model revealed that while the point estimate decreased in the common-effect model (MD = 4.83, 95% CI: 4.46 to 5.20), it remained statistically significant. Given the high heterogeneity, the random-effects model is considered the more appropriate estimate, but the significance holds across both approaches.

Publication Bias and Certainty of Evidence

**Figure 11:**
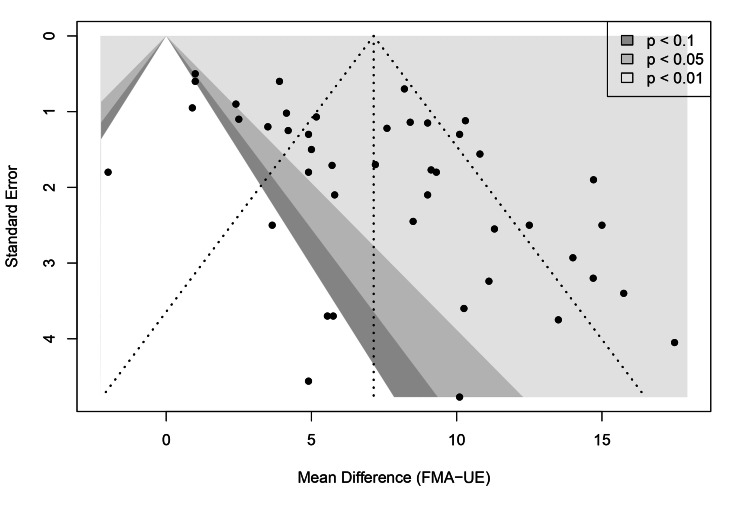
Contour-enhanced funnel plot for the primary outcome (FMA-UE). The shaded regions represent levels of statistical significance. Abbreviation: FMA-UE: Fugl-Meyer Assessment for upper extremity

**Figure 12:**
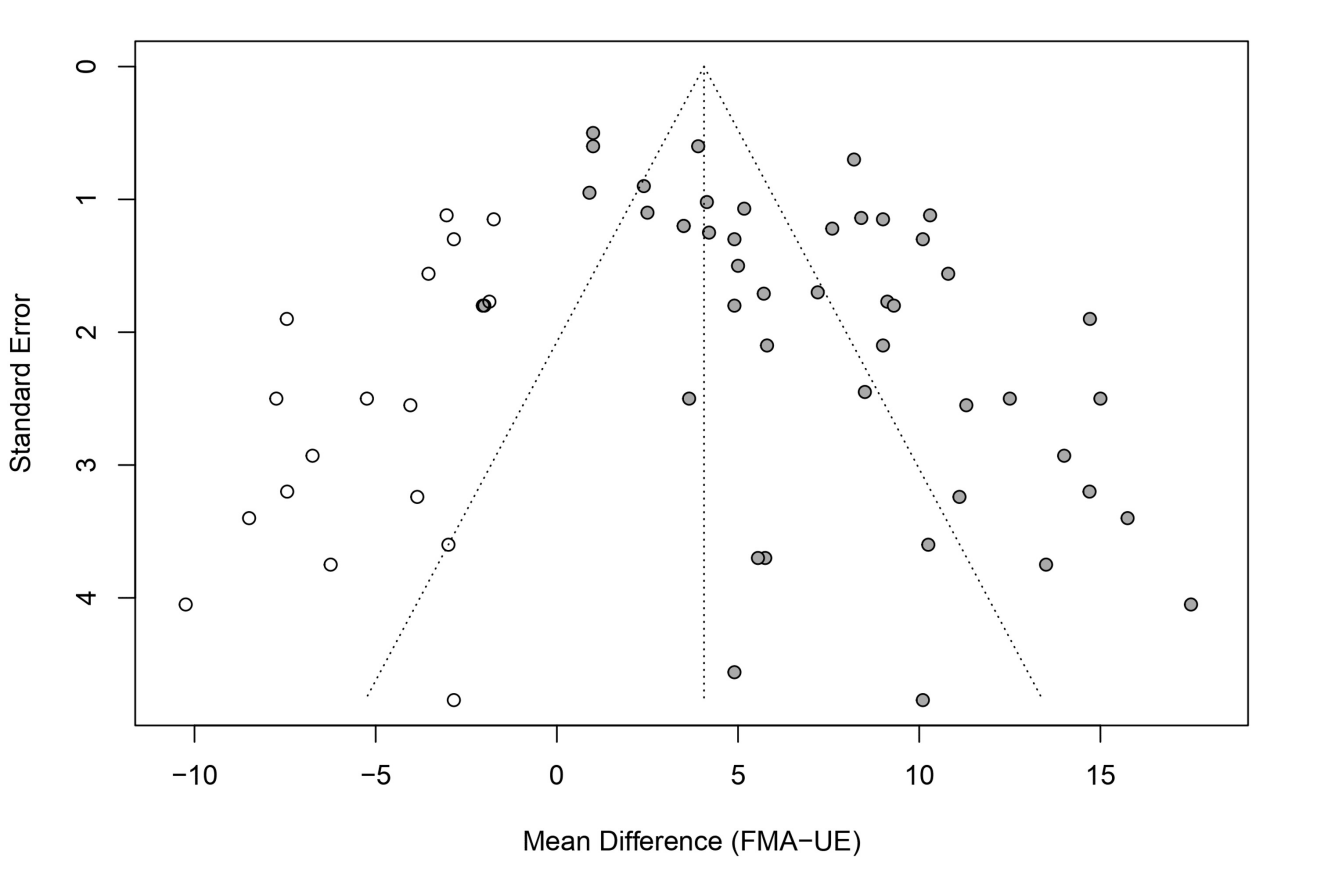
Funnel plot with trim-and-fill adjustment. Open circles represent observed studies; filled circles represent imputed missing studies.

Publication bias: Publication bias was visually assessed using a contour-enhanced funnel plot (Figure 11). The plot displayed some asymmetry, with a gap in the lower left quadrant, suggesting the potential absence of small, non-significant studies. However, Egger’s linear regression test did not detect significant asymmetry (t = 1.86, p = 0.10), indicating that small study effects were not statistically evident. To further probe the robustness of the results, a trim-and-fill analysis was performed. This method imputed 18 potentially missing studies to symmetrize the funnel plot. The adjusted pooled effect size decreased to 4.06 (95% CI: 2.35 to 5.78) but remained statistically significant (p < 0.0001), suggesting that even if publication bias exists, it does not negate the overall finding of a beneficial effect (Figure 12).

Certainty of evidence (GRADE): The overall certainty of the evidence was evaluated using the GRADE approach. The evidence for the primary outcome (FMA-UE) was graded as moderate. The certainty was downgraded by one level for RoB due to the presence of some studies with single-blinding and retrospective registration. However, it was not downgraded for Inconsistency, as heterogeneity was explained by stroke chronicity subgroups, nor for imprecision, given the large total sample size (N = 1,568) and narrow confidence intervals (Table 4). Indirectness was not a concern, as all studies directly compared tDCS to sham in the target population. Publication Bias was not serious based on the robustness of the trim-and-fill analysis.

**Table 4:** Summary of findings and GRADE certainty of evidence assessment. Abbreviations: CI: confidence interval; MD: mean difference; SMD: standardized mean difference; RR: risk ratio; MCID: minimal clinically important difference; RCT: randomized controlled trial, GRADE: Grading of Recommendations Assessment, Development, and Evaluation

Outcome	No. of participants (studies)	Absolute effect (95% CI)	Relative effect (95% CI)	Certainty of evidence (GRADE)	Comments
Upper extremity motor impairment (FMA-UE)	1,568 (44 RCTs)	MD 7.11 (5.76 to 8.46)	—	⨁⨁⨁◯ MODERATE	Downgraded once for risk of bias (single blinding in some trials). Effect exceeds MCID.
Functional activity (ARAT, WMFT, BBT)	771 (17 RCTs)	SMD 0.62 (0.48 to 0.76)	—	⨁⨁⨁◯ MODERATE	Downgraded once for risk of bias. Represents a medium-to-large clinical effect.
Adverse events / safety	590 (10 RCTs)	—	RR 0.96 (0.59 to 1.58)	⨁⨁⨁◯ MODERATE	Downgraded once for imprecision (wide CI crossing unity). No significant difference between active and sham.

**Figure 13:**
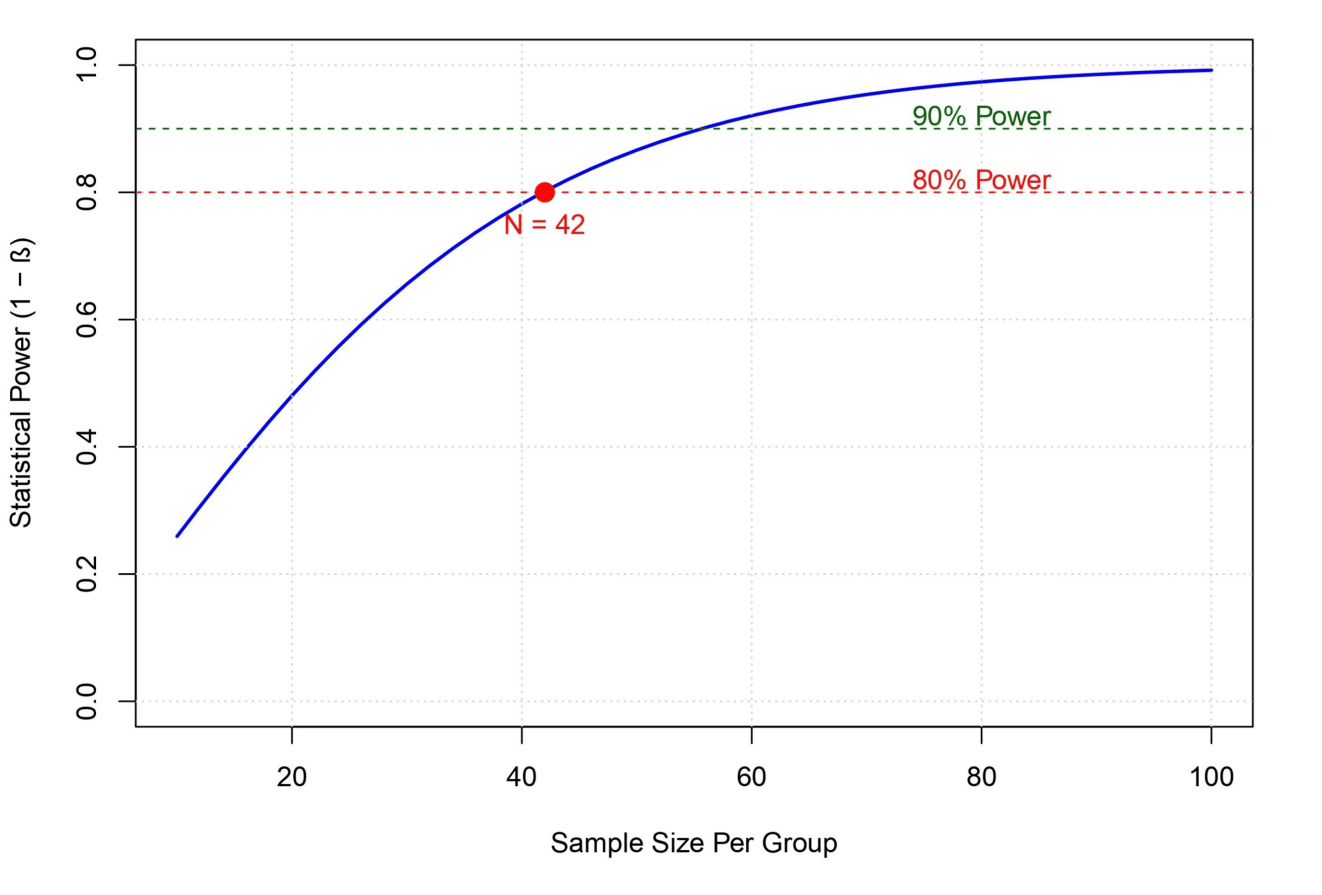
Power analysis for future tDCS trials. The curve illustrates the relationship between sample size per group and statistical power, based on the pooled effect size (d = 0.62) observed for functional activity. The red point indicates that 42 participants per group are necessary to achieve 80% power (1–β = 0.80). Abbreviation: tDCS: transcranial direct current stimulation

Sample size estimation: Based on the pooled effect size observed for functional activity (SMD = 0.62), a post-hoc power analysis was conducted to guide future research. The analysis indicates that a sample size of 42 participants per group is required to achieve 80% statistical power at an alpha level of 0.05 (Figure 13).

Discussion

This systematic review and meta-analysis synthesised data from 45 RCTs involving 1,568 participants to evaluate the efficacy and safety of tDCS combined with rehabilitation for upper extremity motor recovery after a stroke. The results provide robust evidence that active tDCS, whether anodal, cathodal, or bihemispheric, significantly enhances motor impairment recovery compared to sham stimulation, as measured by the FMA-UE. The pooled mean difference of 7.11 points exceeded the MCID of five to 10 points established for this population [10], indicating a tangible clinical benefit. Furthermore, significant improvements were observed in functional activity measures (SMD = 0.62), suggesting that motor gains translate to better performance in daily tasks. Importantly, safety analyses confirmed that tDCS was well tolerated, with no significant increase in adverse events compared to the sham group.

Efficacy and Critical Windows for Recovery

Subgroup analysis revealed that the timing of the intervention played a crucial role in its efficacy. The most pronounced effects were observed in the subacute phase (one to six months post-stroke), with a mean difference of 11.72 points, compared with smaller gains in the chronic phase (4.89 points). This finding aligns with the concept of a "critical window" for neuroplasticity, in which the brain is most responsive to remodelling and synaptic strengthening [68,69]. While chronic patients still benefited, the magnitude of recovery was lower, supporting the recommendation to prioritise neuromodulation interventions earlier in the rehabilitation trajectory [70].

Stimulation Parameters and Dosage

Meta-regression analysis identified current density as a significant moderator of efficacy, with higher current densities associated with greater motor improvement. This supports recent trends in non-invasive brain stimulation research advocating for optimized dosing to overcome inter-individual variability in skull thickness and brain anatomy [71]. Conversely, the total treatment duration (minutes) did not significantly predict outcomes, suggesting that simply extending the duration of stimulation may not yield additional benefits without optimizing other parameters, such as intensity or montage. This finding challenges the "more is better" assumption and highlights the need for dose-finding studies to determine the optimal stimulation duration [72].

Comparison of Montages

The analysis found no significant difference in efficacy between the anodal, cathodal, and bihemispheric montages. This contrasts with some theoretical models suggesting that bihemispheric stimulation might be superior by addressing interhemispheric imbalance (rebalancing transcallosal inhibition) [73]. The lack of superiority for any single montage suggests that the mechanism of action, whether facilitating the ipsilesional cortex or inhibiting the contralesional cortex, may be less critical than the overall modulation of the motor network, or that patient-specific factors (e.g., lesion location, structural reserve) may dictate which montage is most effective for an individual [74,75].

Strengths

This review has several strengths, including a comprehensive search strategy, rigorous RoB assessment using RoB 2, and the use of robust statistical methods (e.g., Hartung-Knapp adjustment, prediction intervals). The inclusion of recent large-scale trials, such as TRANSPORT2 [59] and the NETS trial [62], significantly enhanced the statistical power and precision of the current estimates compared with previous meta-analyses [76,77].

Limitations

This study has some limitations. First, substantial statistical heterogeneity was observed (I_2_ = 88.8%), reflecting the diverse clinical populations and protocols used across the studies. Although subgroup analyses explained some of this variance, considerable unexplained heterogeneity remained. Second, approximately 40% of studies raised "Some Concerns" regarding RoB, due to single-blinding designs or lack of pre-registered protocols. Although sensitivity analyses excluding these studies confirmed the robustness of the primary findings, future trials should adhere to the CONSORT guidelines to minimize bias. Also, while meta-regression indicated an association with current density, this finding needs caution due to the heterogeneous electrode configurations (e.g., varying sponge sizes, shapes, and positions) across included trials, which may differentially influence the effective electric field reaching the cortex. Finally, long-term follow-up data were limited, preventing firm conclusions regarding the durability of the observed effects beyond the immediate post-intervention period.

Implications for Clinical Practice and Future Research

These findings suggest that tDCS can be a beneficial adjunct to upper limb rehabilitation, particularly in the subacute phase of stroke. Clinicians may consider incorporating tDCS into rehabilitation protocols to enhance motor recovery while adhering to safety guidelines [71].

Future research should focus on investigating whether individualizing montages based on lesion location or connectivity (e.g., using DTI or fMRI) improves outcomes (Stinear et al., 2007), conducting dose-response studies to define the optimal current density and duration, and designing trials with extended follow-up periods (six months to one year) to assess the retention of motor gains. Additionally, to ensure robust statistical validity and avoid Type II errors, future definitive trials should aim for a minimum sample size of 42 participants per group, as indicated by our power analysis based on the observed functional effect size of 0.62. Research should utilize neurophysiological measures (e.g., TMS-MEP) to better understand the neural correlates of recovery and identify responders versus non-responders.

## Conclusions

This systematic review and meta-analysis provides robust evidence that tDCS is an effective adjunct to rehabilitation for improving upper extremity motor function in stroke survivors. The analysis demonstrated a statistically significant improvement in FMA-UE scores, exceeding the minimal clinically important difference, particularly when the intervention is applied in the subacute phase of recovery. However, because of the substantial statistical heterogeneity observed across the included trials, these results should be interpreted with caution regarding their application to individual cases. The wide prediction intervals indicate that while the average therapeutic effect is positive, clinical outcomes may vary significantly based on patient-specific factors and the precise stimulation parameters employed. Moreover, functional activity showed moderate improvement, but the benefits were most pronounced in motor impairment reduction. The intervention was found to be safe, with no significant increase in adverse events compared to sham stimulation. These findings support the integration of tDCS into stroke rehabilitation protocols, specifically emphasizing early intervention and optimized current densities, while highlighting the need for future research to standardize stimulation parameters.

## References

[1] Awosika OO, Cohen LG (2019). Transcranial Direct Current Stimulation in Stroke Rehabilitation: Present and Future. Practical guide to transcranial direct current stimulation.

[2] Simis M, Morales L, Marduy A, Fregni F (2021). tDCS in the Context of Rehabilitation. Transcranial direct current stimulation in neuropsychiatric disorders.

[3] Reato D, Salvador R, Bikson M, Opitz A, Dmochowski J, Miranda PC (2019). Principles of Transcranial Direct Current Stimulation (tDCS): Introduction to the Biophysics of tDCS. Practical guide to transcranial direct current stimulation.

[4] Rossini PM, Burke D, Chen R (2015). Non-invasive electrical and magnetic stimulation of the brain, spinal cord, roots and peripheral nerves: Basic principles and procedures for routine clinical and research application. An updated report from an I.F.C.N. Committee. Clin Neurophysiol.

[5] Woods AJ, Martin DM (2021). Clinical Research and Methodological Aspects for tDCS Research. Transcranial direct current stimulation in neuropsychiatric disorders.

[6] Uswatte G, Taub E, Morris D, Light K, Thompson PA (2006). The Motor Activity Log-28: assessing daily use of the hemiparetic arm after stroke. Neurology.

[7] Page MJ, McKenzie JE, Bossuyt PM (2021). The PRISMA 2020 statement: an updated guideline for reporting systematic reviews. BMJ.

[8] Baraah AO, Faisal A, Bayan HMK (2025). Effect of transcranial direct current stimulation combined with rehabilitation on arm and hand function in stroke patients: a systematic review and meta-analysis. PROSPERO.

[9] Higgins JPT, Thomas J, Chandler J (2019). Cochrane handbook for systematic reviews of interventions. Cochrane handbook for systematic reviews of interventions. 2nd ed. Chichester (UK): John Wiley & Sons.

[10] Gladstone DJ, Danells CJ, Black SE (2002). The fugl-meyer assessment of motor recovery after stroke: a critical review of its measurement properties. Neurorehabil Neural Repair.

[11] Yozbatiran N, Der-Yeghiaian L, Cramer SC (2008). A standardized approach to performing the action research arm test. Neurorehabil Neural Repair.

[12] Wolf SL, Catlin PA, Ellis M, Archer AL, Morgan B, Piacentino A (2001). Assessing Wolf motor function test as outcome measure for research in patients after stroke. Stroke.

[13] Mathiowetz V, Volland G, Kashman N, Weber K (1985). Adult norms for the Box and Block Test of manual dexterity. Am J Occup Ther.

[14] Sterne JA, Savović J, Page MJ (2019). RoB 2: a revised tool for assessing risk of bias in randomised trials. BMJ.

[15] Schwarzer G, Carpenter JR, Rücker G (2015). Meta-analysis with R. Meta-analysis with R.

[16] Borenstein M, Hedges LV, Higgins JPT, Rothstein HR (2021). Introduction to meta-analysis. Hoboken (NJ): Wiley.

[17] Huedo-Medina TB, Sánchez-Meca J, Marín-Martínez F, Botella J (2006). Assessing heterogeneity in meta-analysis: Q statistic or I2 index?. Psychol Methods.

[18] Sterne JAC, Becker BJ, Egger M (2005). The Funnel Plot. Publication Bias in Meta-Analysis: Prevention, Assessment and Adjustments.

[19] Egger M, Davey Smith G, Schneider M, Minder C (1997). Bias in meta-analysis detected by a simple, graphical test. BMJ.

[20] Sterne JAC, Egger M (2005). Regression Methods to Detect Publication and Other Bias in Meta-Analysis. Publication bias in meta-analysis: prevention, assessment and adjustments.

[21] Duval S (2005). The Trim and Fill Method. Publication bias in meta-analysis: prevention, assessment and adjustments.

[22] Cohen J (1988). Statistical power analysis for the behavioral sciences.

[23] Alisar DC, Ozen S, Sozay S (2020). Effects of bihemispheric transcranial direct current stimulation on upper extremity function in stroke patients: a randomized double-blind sham-controlled study. J Stroke Cerebrovasc Dis.

[24] Allman C, Amadi U, Winkler AM (2016). Ipsilesional anodal tDCS enhances the functional benefits of rehabilitation in patients after stroke. Sci Transl Med.

[25] Ang KK, Guan C, Phua KS (2015). Facilitating effects of transcranial direct current stimulation on motor imagery brain-computer interface with robotic feedback for stroke rehabilitation. Arch Phys Med Rehabil.

[26] Beaulieu LD, Blanchette AK, Mercier C, Bernard-Larocque V, Milot MH (2019). Efficacy, safety, and tolerability of bilateral transcranial direct current stimulation combined to a resistance training program in chronic stroke survivors: a double-blind, randomized, placebo-controlled pilot study. Restor Neurol Neurosci.

[27] Bernal-Jiménez JJ, Dileone M, Mordillo-Mateos L (2024). Combining transcranial direct current stimulation with hand robotic rehabilitation in chronic stroke patients: a double-blind randomized clinical trial. Am J Phys Med Rehabil.

[28] Bornheim S, Croisier JL, Maquet P, Kaux JF (2020). Transcranial direct current stimulation associated with physical-therapy in acute stroke patients - a randomized, triple blind, sham-controlled study. Brain Stimul.

[29] Chen J, Du J, Wang C, Yu H (2025). Cathodal tDCS and robotic therapy for upper limb rehabilitation in chronic stroke: a randomized controlled trial. Front Neurol.

[30] Cho HS, Cha HG (2015). Effect of mirror therapy with tDCS on functional recovery of the upper extremity of stroke patients. J Phys Ther Sci.

[31] Dehem S, Gilliaux M, Lejeune T (2018). Effectiveness of a single session of dual-transcranial direct current stimulation in combination with upper limb robotic-assisted rehabilitation in chronic stroke patients: a randomized, double-blind, cross-over study. Int J Rehabil Res.

[32] Figlewski K, Blicher JU, Mortensen J, Severinsen KE, Nielsen JF, Andersen H (2017). Transcranial direct current stimulation potentiates improvements in functional ability in patients with chronic stroke receiving constraint-induced movement therapy. Stroke.

[33] Gao L, Chu F, Liu X, Chen J, Zhang M, Zhang Y (2025). Effects of occupational therapy synchronized with dual transcranial direct current stimulation on upper limb function and electroencephalography power in subacute stroke patients: a randomized, double-blind, controlled study. PLoS One.

[34] Garrido M M, Álvarez E E, Acevedo P F, Moyano V Á, Castillo N N, Cavada Ch G (2023). Early transcranial direct current stimulation with modified constraint-induced movement therapy for motor and functional upper limb recovery in hospitalized patients with stroke: a randomized, multicentre, double-blind, clinical trial. Brain Stimul.

[35] Guo C, Geng A, Sui Y (2025). Effect of tDCS concurrent with VR-based robotic intervention on hemiplegic upper limb function after subacute ischemic stroke: a randomized controlled study. Neural Plast.

[36] Hesse S, Waldner A, Mehrholz J, Tomelleri C, Pohl M, Werner C (2011). Combined transcranial direct current stimulation and robot-assisted arm training in subacute stroke patients: an exploratory, randomized multicenter trial. Neurorehabil Neural Repair.

[37] Hsu SP, Lu CF, Lin BF (2023). Effects of bihemispheric transcranial direct current stimulation on motor recovery in subacute stroke patients: a double-blind, randomized sham-controlled trial. J Neuroeng Rehabil.

[38] Kashoo FZ, Al-Baradie RS, Alzahrani M (2022). Effect of transcranial direct current stimulation augmented with motor imagery and upper-limb functional training for upper-limb stroke rehabilitation: a prospective randomized controlled trial. Int J Environ Res Public Health.

[39] Kim DY, Lim JY, Kang EK, You DS, Oh MK, Oh BM, Paik NJ (2010). Effect of transcranial direct current stimulation on motor recovery in patients with subacute stroke. Am J Phys Med Rehabil.

[40] Koh CL, Lin JH, Jeng JS, Huang SL, Hsieh CL (2017). Effects of transcranial direct current stimulation with sensory modulation on stroke motor rehabilitation: a randomized controlled trial. Arch Phys Med Rehabil.

[41] Lee DG, Lee DY (2015). Effects of adjustment of transcranial direct current stimulation on motor function of the upper extremity in stroke patients. J Phys Ther Sci.

[42] Lee SJ, Chun MH (2014). Combination transcranial direct current stimulation and virtual reality therapy for upper extremity training in patients with subacute stroke. Arch Phys Med Rehabil.

[43] Li C, Chen Y, Tu S (2024). Dual-tDCS combined with sensorimotor training promotes upper limb function in subacute stroke patients: a randomized, double-blinded, sham-controlled study. CNS Neurosci Ther.

[44] Liao WW, Chiang WC, Lin KC (2020). Timing-dependent effects of transcranial direct current stimulation with mirror therapy on daily function and motor control in chronic stroke: a randomized controlled pilot study. J Neuroeng Rehabil.

[45] Liao WW, Lin CY, Horng YS, Chen CL, Lee TH, Wu CY (2025). Transcranial direct current stimulation over the motor and premotor cortex with mirror therapy improves motor control, muscle function, and brain activity in chronic stroke: a double-blind randomized sham-controlled trial. J Neuroeng Rehabil.

[46] Lindenberg R, Renga V, Zhu LL, Nair D, Schlaug G (2010). Bihemispheric brain stimulation facilitates motor recovery in chronic stroke patients. Neurology.

[47] Lindenberg R, Zhu LL, Schlaug G (2012). Combined central and peripheral stimulation to facilitate motor recovery after stroke: the effect of number of sessions on outcome. Neurorehabil Neural Repair.

[48] Mazzoleni S, Tran VD, Iardella L, Dario P, Posteraro F (2017). Randomized, sham-controlled trial based on transcranial direct current stimulation and wrist robot-assisted integrated treatment on subacute stroke patients: intermediate results. IEEE Int Conf Rehabil Robot.

[49] Menezes IS, Cohen LG, Mello EA (2018). Combined brain and peripheral nerve stimulation in chronic stroke patients with moderate to severe motor impairment. Neuromodulation.

[50] Morone G, Capone F, Iosa M (2022). May dual transcranial direct current stimulation enhance the efficacy of robot-assisted therapy for promoting upper limb recovery in chronic stroke?. Neurorehabil Neural Repair.

[51] Mortensen J, Figlewski K, Andersen H (2016). Combined transcranial direct current stimulation and home-based occupational therapy for upper limb motor impairment following intracerebral hemorrhage: a double-blind randomized controlled trial. Disabil Rehabil.

[52] Nair DG, Renga V, Lindenberg R, Zhu L, Schlaug G (2011). Optimizing recovery potential through simultaneous occupational therapy and non-invasive brain-stimulation using tDCS. Restor Neurol Neurosci.

[53] Palimeris S, Ansari Y, Remaud A, Tremblay F, Corriveau H, Boudrias MH, Milot MH (2022). Effect of a tailored upper extremity strength training intervention combined with direct current stimulation in chronic stroke survivors: a randomized controlled trial. Front Rehabil Sci.

[54] Pires R, Baltar A, Sanchez MP, Antonino GB, Brito R, Berenguer-Rocha M, Monte-Silva K (2023). Do higher transcranial direct current stimulation doses lead to greater gains in upper limb motor function in post-stroke patients?. Int J Environ Res Public Health.

[55] Rabadi MH, Aston CE (2017). Effect of transcranial direct current stimulation on severely affected arm-hand motor function in patients after an acute ischemic stroke: a pilot randomized control trial. Am J Phys Med Rehabil.

[56] Rocha S, Silva E, Foerster Á (2016). The impact of transcranial direct current stimulation (tDCS) combined with modified constraint-induced movement therapy (mCIMT) on upper limb function in chronic stroke: a double-blind randomized controlled trial. Disabil Rehabil.

[57] Salazar AP, Cimolin V, Schifino GP, Rech KD, Marchese RR, Pagnussat AS (2020). Bi-cephalic transcranial direct current stimulation combined with functional electrical stimulation for upper-limb stroke rehabilitation: a double-blind randomized controlled trial. Ann Phys Rehabil Med.

[58] Sattler V, Acket B, Raposo N (2015). Anodal tDCS combined with radial nerve stimulation promotes hand motor recovery in the acute phase after ischemic stroke. Neurorehabil Neural Repair.

[59] Schlaug G, Cassarly C, Feld JA (2025). Safety and efficacy of transcranial direct current stimulation in addition to constraint-induced movement therapy for post-stroke motor recovery (TRANSPORT2): a phase 2, multicentre, randomised, sham-controlled triple-blind trial. Lancet Neurol.

[60] Shaheiwola N, Zhang B, Jia J, Zhang D (2018). Using tDCS as an add-on treatment prior to FES therapy in improving upper limb function in severe chronic stroke patients: a randomized controlled study. Front Hum Neurosci.

[61] Straudi S, Fregni F, Martinuzzi C, Pavarelli C, Salvioli S, Basaglia N (2016). tDCS and robotics on upper limb stroke rehabilitation: effect modification by stroke duration and type of stroke. Biomed Res Int.

[62] (2024). Efficacy and safety of transcranial direct current stimulation to the ipsilesional motor cortex in subacute stroke (NETS): a multicenter, randomized, double-blind, placebo-controlled trial. Lancet Reg Health Eur.

[63] Triccas LT, Burridge JH, Hughes A, Verheyden G, Desikan M, Rothwell J (2015). A double-blinded randomised controlled trial exploring the effect of anodal transcranial direct current stimulation and uni-lateral robot therapy for the impaired upper limb in sub-acute and chronic stroke. NeuroRehabilitation.

[64] Viana RT, Laurentino GE, Souza RJ (2014). Effects of the addition of transcranial direct current stimulation to virtual reality therapy after stroke: a pilot randomized controlled trial. NeuroRehabilitation.

[65] Wu D, Qian L, Zorowitz RD, Zhang L, Qu Y, Yuan Y (2013). Effects on decreasing upper-limb poststroke muscle tone using transcranial direct current stimulation: a randomized sham-controlled study. Arch Phys Med Rehabil.

[66] Yao X, Cui L, Wang J, Feng W, Bao Y, Xie Q (2020). Effects of transcranial direct current stimulation with virtual reality on upper limb function in patients with ischemic stroke: a randomized controlled trial. J Neuroeng Rehabil.

[67] Zhang X, Meesen R, Swinnen SP, Feys H, Woolley DG, Cheng HJ, Wenderoth N (2024). Combining muscle-computer interface guided training with bihemispheric tDCS improves upper limb function in patients with chronic stroke. J Neurophysiol.

[68] Krakauer JW, Carmichael ST, Corbett D, Wittenberg GF (2012). Getting neurorehabilitation right: what can be learned from animal models?. Neurorehabil Neural Repair.

[69] Zeiler SR, Krakauer JW (2013). The interaction between training and plasticity in the poststroke brain. Curr Opin Neurol.

[70] Bernhardt J, Godecke E, Johnson L, Langhorne P (2017). Early rehabilitation after stroke. Curr Opin Neurol.

[71] Bikson M, Grossman P, Thomas C (2016). Safety of transcranial direct current stimulation: evidence based update. Brain Stimul.

[72] Nitsche MA, Paulus W (2000). Excitability changes induced in the human motor cortex by weak transcranial direct current stimulation. J Physiol.

[73] Di Pino G, Pellegrino G, Assenza G (2014). Modulation of brain plasticity in stroke: a novel model for neurorehabilitation. Nat Rev Neurol.

[74] Grefkes C, Ward NS (2014). Cortical reorganization after stroke: how much and how functional?. Neuroscientist.

[75] Stinear CM, Barber PA, Smale PR, Coxon JP, Fleming MK, Byblow WD (2007). Functional potential in chronic stroke patients depends on corticospinal tract integrity. Brain.

[76] Elsner B, Kugler J, Pohl M, Mehrholz J (2020). Transcranial direct current stimulation (tDCS) for improving activities of daily living, and physical and cognitive functioning, in people after stroke. Cochrane Database Syst Rev.

[77] Marquez J, van Vliet P, McElduff P, Lagopoulos J, Parsons M (2015). Transcranial direct current stimulation (tDCS): does it have merit in stroke rehabilitation? A systematic review. Int J Stroke.

